# The Use of Analgesics during Vaccination with a Live Attenuated *Yersinia pestis* Vaccine Alters the Resulting Immune Response in Mice

**DOI:** 10.3390/vaccines7040205

**Published:** 2019-12-03

**Authors:** Marilynn J. Culbreth, Sergei S. Biryukov, Jennifer L. Shoe, Jennifer L. Dankmeyer, Melissa Hunter, Christopher P. Klimko, Raysa Rosario-Acevedo, David P. Fetterer, Alicia M. Moreau, Susan L. Welkos, Christopher K. Cote

**Affiliations:** 1 United States Army Medical Research Institute of Infectious Diseases (USAMRIID), Comparative Medicine Division, Fort Detrick, Frederick, MD 21702, USA; marilynn.j.culbreth.mil@mail.mil; 2 United States Army Medical Research Institute of Infectious Diseases (USAMRIID), Bacteriology Division, Fort Detrick, Frederick, MD 21702, USA; sergei.s.biryukov.mil@mail.mil (S.S.B.); jennifer.l.shoe.ctr@mail.mil (J.L.S.); jennifer.l.dankmeyer.ct@mail.mil (J.L.D.); melissa.hunter.ctr@mail.mil (M.H.); christopher.p.klimko2.ctr@mail.mil (C.P.K.); raysa.rosarioacevedo.mil@mail.mil (R.R.-A.); susan.l.welkos.civ@mail.mil (S.L.W.); 3 United States Army Medical Research Institute of Infectious Diseases (USAMRIID), Biostatistics Medicine Division, Fort Detrick, Frederick, MD 21702, USA; david.p.fetterer.ctr@mail.mil; 4 United States Army Medical Research Institute of Infectious Diseases (USAMRIID), Pathology Division, Fort Detrick, Frederick, MD 21702, USA; alicia.m.moreau.mil@mail.mil

**Keywords:** *Yersinia pestis*, plague, live vaccine, mice, immune response, analgesia, acetaminophen, meloxicam

## Abstract

The administration of antipyretic analgesics prior to, in conjunction with, or due to sequelae associated with vaccination is a common yet somewhat controversial practice. In the context of human vaccination, it is unclear if even short-term analgesic regimens can significantly alter the resulting immune response, as literature exists to support several scenarios including substantial immune interference. In this report, we used a live attenuated *Yersinia pestis* vaccine to examine the impact of analgesic administration on the immune response elicited by a single dose of a live bacterial vaccine in mice. Mice were assessed by evaluating natural and provoked behavior, as well as food and water consumption. The resulting immune responses were assessed by determining antibody titers against multiple antigens and assaying cellular responses in stimulated splenocytes collected from vaccinated animals. We observed no substantial benefit to the mice associated with the analgesic administration. Splenocytes from both C57BL/6 and BALB/c vaccinated mice receiving acetaminophen have a significantly reduced interferon-gamma (IFN-γ) recall response. Additionally, there is a significantly lower immunoglobulin (Ig)G2a/IgG1 ratio in vaccinated BALB/c mice treated with either acetaminophen or meloxicam and a significantly lower IgG2c/IgG1 ratio in vaccinated C57BL/6 mice treated with acetaminophen. Taken together, our data indicate that the use of analgesics, while possibly ethically warranted, may hinder the accurate characterization and evaluation of novel vaccine strategies with little to no appreciable benefits to the vaccinated mice.

## 1. Introduction

The administration of antipyretic analgesics is now a routine practice for patients receiving vaccinations and is recommended to ameliorate local and systemic side effects like fever and pain [1,2]. Analgesics such as acetaminophen, meloxicam, or ibuprofen are used as preventive strategies to reduce vaccine-related reactogenicity initiated at the time of vaccine administration and/or post-vaccination [2]. Adverse vaccination reactions such as localized swelling, rash, pain, and systemic reactions of fever, muscle pain, vomiting or diarrhea, and in severe cases central nervous system effects may entice some patients to use analgesics with or without medical directives in conjunction with vaccinations. These reactions usually occur within 24–48 h following the vaccination. However, while antipyretic analgesics were shown to decrease side effects following vaccine administration, evidence exists detailing possible deleterious effects that acetaminophen or other analgesics may have on the immune response generated by different vaccine antigens [3,4].

Prymula and colleagues assessed the effects of prophylactic administration of acetaminophen (paracetamol) and compared changes in fever and vaccine-specific antibody titers in immunized children receiving analgesics with other immunized children not receiving analgesics. Results showed that fever was significantly reduced in children that were given analgesic, but antibody titers were also significantly lower when compared with children that did not receive analgesia. However, a follow-up study in 2013 by Prymula et al. evaluated the effect of acetaminophen on the long-term persistence and boosting of antibody in response to a multi-valent pneumococcal non-typeable *Haemophilus influenzae* protein D conjugate vaccine [5]. These data demonstrated that the acetaminophen group had lower antibody titers prior to the boost, but both groups had a similar robust increase in titers following the boost [5]. The immune blunting that was observed in the initial study was not observed after a booster vaccination, suggesting that antipyretic analgesics may not affect memory B cells. The mechanism of antipyretic analgesic action on the immune response remains unclear. Whaley and Sloane demonstrated that anti-inflammatory drugs can inhibit the complement system without altering the antibody binding [6]. Antipyretic analgesics may impact the adaptive immune response at the cellular level affecting the entire process through antibody production [2]. The existing human data are somewhat contradictory and may be skewed depending upon the type of vaccination employed in the trials.

While vaccinations are generally expected to only cause minor pain upon injection that may resolve soon after the procedure, there are vaccines and regimens that can cause pain or distress in research animals. For example, differential adjuvanation may be a source of increased pain or distress. In a study evaluating complete Freund’s adjuvant (CFA) or incomplete Freund’s adjuvant (IFA) immunization with mice, there was appreciable pain with these products [7]. Live attenuated vaccines offer other examples of highly successful, but potentially more reactogenic vaccine strategies [8,9]. These vaccines are delivered as attenuated infections and rely on interactions of the live vaccine and the host immune system to develop a protective immune response. While the immunity elicited by live attenuated vaccines is often associated with more robust cellular immunity than what is seen in subunit vaccines, these vaccines could be more painful/stressful by promoting an active infection [9,10,11].

Acetaminophen is frequently recommended for its antipyretic properties during infant vaccinations to prevent convulsions due to high fevers and, unlike aspirin, there is little risk of sensitivity reactions [3,12,13,14,15]. Acetaminophen was shown to have little potential for abuse or toxicity, and it lacks antiplatelet activity and gastro-toxicity seen with other non-steroidal anti-inflammatory drugs (NSAIDs) [16]. Acetaminophen lacks the anti-inflammatory properties seen with cyclooxygenase-2 (COX-2) inhibitors; however, anti-inflammatory activity is associated with high doses of acetaminophen, at which point there may be risk of hepato-toxicity [3,12,13,16,17,18,19,20]. Acetaminophen is not considered a selective COX-2 inhibitor nor is it completely understood if it impacts immune stimulation during live vaccination [7,15,21]. A diminished immune response was observed when acetaminophen was prophylactically given during immunization as measured by antibody titers in some studies [3,4,22], but other studies noted no significant difference [7]. The mechanism of action of acetaminophen is not completely elucidated but it may inhibit the cyclooxygenase pathway as a selective COX-3 inhibitor [18,23,24]. The effectiveness of acetaminophen to alleviate pain in rodents also remains in question [25,26,27,28,29,30,31,32,33,34,35].

Meloxicam acts by inhibiting COX-2 and prostaglandin synthesis and is routinely given subcutaneously. There is conflicting information in the literature about the effect of meloxicam on immunity. COX-2 is described as crucial in both the innate and the adaptive immune response [7,36]. Meloxicam does not appear to affect acute-phase proteins and specific antibody titers to *Escherichia coli* lipopolysaccharide in a porcine model [37]. In 2014, Das et al. performed a systematic review of antipyretic administration during the post-vaccination period and determined that, even with the reduction in fever and suppression of the antibody response, there was no overt interference with disease prevention [12]. However, one report showed a statistically significant increase in antibody titers in the presence of meloxicam [7]. Analgesics such as acetaminophen, meloxicam, and buprenorphine were recommended after a study employing complete and incomplete Freund’s adjuvant immunization of mice to alleviate pain without impacting antibody production [7]. During a study with *Listeria monocytogenes*-based immunotherapy, COX-1 was found to be detrimental, but COX-2 was determined to be critical for immunity to *L. monocytogenes* [15]. Another study demonstrated that COX-2 inhibitors reduced neutralizing antibodies, allowing for repeated administration of vaccinia virus for control of ovarian cancer [38].

We evaluated the immune response elicited by a live attenuated *Yersinia pestis* vaccine when the analgesics acetaminophen (paracetamol) or meloxicam were given to BALB/c or C57BL/6 mice. *Y. pestis*, a facultative intracellular bacterium primarily found in wild rodents, in parts of Africa, Asia, and North America, is the etiologic agent of plague [39,40,41,42]. Infection with *Y. pestis* can manifest in three forms of the disease: pneumonic, septicemic, and bubonic. The most common form is bubonic plague, characterized by a swollen lymph node referred to as a bubo [43,44]. From a biodefense perspective, pneumonic plague remains a scenario of concern due to person-to-person transmission and a low infectious dose [45]. Plague vaccines, including live vaccine strategies at various stages of development, are being investigated [46,47,48,49]. We hypothesized that antipyretic analgesics, specifically acetaminophen and meloxicam, would alter the immune response in mice if used during vaccination with a live bacterial vaccine.

## 2. Materials and Methods

### 2.1. Bacteria

We used fully virulent *Y. pestis* CO92 [50], a non-encapsulated mutant of CO92 referred to as C12 [51], and the live attenuated *pgm*^−^/pPst^−^ mutant of CO92 referred to as VAX [52,53]. Both *Y. pestis* CO92 and the C12 derivative are fully virulent tier-one select agents that require Biological Safety Level-3 (BSL-3) laboratory conditions. The *Y. pestis* VAX strain is an exempt strain and can safely be worked with at BSL-2 laboratory conditions [54]. Before use as either ELISA capture antigens or splenocyte stimulants, bacterial cells were subjected to approximately 21 kGy of γ-radiation using JL Shepherd irradiator model 109-68.

### 2.2. Animals

For vaccination, we used 180 mice from Charles River Laboratories (Frederick, MD): 90 female C57BL/6 mice and 90 female BALB/c mice (age, 6–8 weeks; weight, approximately 17–18 g). The study design and all procedures were approved by the United States Army Medical Research Institute of Infectious Diseases Institutional Animal Care and Use Committee (IACUC). Housing parameters were in accordance with the Guide for the Care and use of Laboratory Animals, in an Association for Assessment and Accreditation of Laboratory Animal Care International (AAALAC)-accredited facility [55]. All mice were socially housed in groupings of 10 in solid-bottom polycarbonate cages (Allentown Caging, Allentown, NJ; Lab Products, Seaford, DE), which were fitted with a static filter-top. Mice were fed a pelleted diet (no. 2018 Teklad Global 18% Protein, Envigo, Frederick, MD), and municipal water (no further treatment) was provided in sipper water bottles with stoppers. To minimize water loss from the drinking bottles, the husbandry and veterinary staff were requested to not handle the racks or cages. For enrichment, the mice were provided compressed cotton squares (Nestlets, Ancare, Bellmore, NY) and paper nesting material (Enviro-Dri, Shepherd Specialty Papers, Watertown, TN), along with polycarbonate igloos/nestlets (BioServ, Flemington, NJ). The cage bedding was cellulose (7070C Teklad Certified Diamond Dry Cellulose Bedding, Envigo, Indianapolis, IN).

### 2.3. Analgesia

Pans of mice were randomized into three groups for each strain (*n* = 20 per group). The groups were designated as BALB/c acetaminophen (ACE) with or without vaccine (VAX), BALB/c meloxicam (MEL) with or without VAX, BALB/c no treatment with or without VAX, C57BL/6 ACE with or without VAX, C57BL/6 MEL with or without VAX, and C57BL/6 no treatment with or without VAX. Mouse, food, and water weights were taken every morning at approximately 1 h after lights on starting on day 2 and ending on day 6. Additionally, mouse weights were recorded on days 7, 9, 11, 18, 25, 32, 42, and 46. Daily water consumption by mice is estimated to be approximately 4–8 mL/30 g mouse [56]. The weights of the food and water were recorded to evaluate consumption rates and assess wellbeing. If the food or water was replenished, the before and after weights were taken. Brunell et al. reported that female C57BL/6 mice consumed approximately 4–6 mL/day of acetaminophen-medicated water [57]. This was in agreement with our baseline water consumption rate which was determined to be approximately 3–5 mL/20 g mouse per day. Water and food consumption rates, along with daily observations during the vaccination period, were used to evaluate changes in behavior.

ACE was delivered ad libitum in water starting on day 0 and replenished on day 1 and day 2 during the vaccination period. The delivery of ACE in water allowed for minimal manipulation of mice, which reduced the amount of stress to the animal and also provided a level of occupational safety within BSL-3 containment laboratories. VAX was given on day 1 allowing for 24 hours of ACE to be provided. ACE is stable in aqueous solution at room temperature and neutral pH [25]. The weight of the water was taken before and after each time the medicated water was replenished. The recommended dose of oral ACE is 110–305 mg/kg [28,58,59]. To achieve 180–300 mg/kg/day for the mice, 7.5 mL of flavored children’s ACE (160 mg/5 mL) was added to 200 mL of water. The ACE dose per mouse was calculated by taking the average amount of water consumed (3–5 mL) multiplied by the concentration (water, 1–2 mg/mL [1.2 mg/mL]) divided by the average weight (kg) of the mice in the pan each day mg/kg/day. Due to availability, on day 0, grape-flavored ACE was used and then, on days 1 and 2, cherry-flavored ACE was used. Due to the duration and sample size, we did not observe any significant flavor-associated changes in water consumption rate. A commonly used dose regimen for MEL in mice is 1–5 mg/kg [59]. For this study, we used a Loxicom (MEL) 5 mg/mL solution for injection from Manufacture Norbrook Laboratories Limited (Newry, BT35 6PU, Co. Down, Northern Ireland). MEL groups received 2 mg/kg (0.2 mL) subcutaneously daily for three days (day 0, day 1, and day 2).

### 2.4. Vaccination

Mice were 7–9 weeks of age at the time of vaccination. Groups of 20 mice were vaccinated in order to euthanize 10 mice for sample collection and downstream immune analyses and 10 mice to proceed with *Y. pestis* challenge. Mice received approximately 1 × 10^7^ colony forming units (CFU) of *Y. pestis pgm*^−^/pPst^−^ live attenuated vaccine strain (VAX) delivered subcutaneously on the central dorsal region in a 0.2-mL volume [52]. For preparation of the vaccination dose, the strain was grown in heart infusion broth (HIB) with 0.2% xylose. Overnight cultures were diluted in HIB with 0.2% xylose and 2.5 mM CaCl_2_ to an optical density(OD_600_) of 0.1 and incubated at 28 °C to an OD_600_ of 0.5–0.8, harvested by centrifugation at 10,000 rpm for 20 min, and suspended in 10 mM potassium phosphate buffered solution (PBS) to a concentration yielding 1 × 10^7^ CFU per dose. Mice were observed daily and scored during the early days after vaccination (days 2–4) to note any impact of analgesia on animal wellbeing (Table 1). Mice were scored on appearance (e.g., coat, eye or nasal discharge, absence of grooming, piloerection, etc.), natural behavior (e.g., less peer interaction, mobility, restlessness, etc.), and provoked behavior (e.g., subdued when stimulated, pre-comatose, etc.). Mice scoring 0–2 were considered normal, mice scoring 3–7 were considered clinically effected and required more frequent observations, and mice scoring ≥8 were considered moribund and were euthanized. Mice were scored in a semi-blinded fashioned by two independent individuals.

### 2.5. Acetaminophen (ACE) Concentrations in Sera

In an attempt to quantify ACE concentration in mice, blood was sampled and ACE serum concentration measurements were taken. In groups of 30 mice, BALB/c or C57BL/6 mice were provided acetaminophen for ad libitum intake with drinking water. Blood was collected from deeply sedated mice (*n* = 10) that were immediately euthanized after the blood collection procedure at 24 h, 48 h, and 72 h after initiation of ACE. Blood was centrifuged and the serum stored at −20 °C until further analysis. ACE serum concentrations were determined by DRI^®^ Acetaminophen-Serum-Tox-Assay Thermo Fisher Scientific, Fremont, CA, USA) by our in-house laboratory. All samples returned a result of <4 μg/mL (below the lower limits of detection for the Vitros 350 slides that were used). Thus, this tox screen platform or sample collection schedule was not suitable for our experiment to monitor acetaminophen delivered ad libitum in drinking water.

### 2.6. Animal Challenges

Twenty-eight days after vaccinations (day 29), some mice were euthanized for sample collection and immunological analyses. On day 29, mice were challenged with fully virulent *Y. pestis* CO92 using the bubonic plague model under Animal Biological Safety Level-3 (ABSL-3) conditions. BALB/c mice received approximately 208 CFU (or approximately 130 lethal dose, 50% (LD_50)_ equivalents), and C57BL/6 mice received approximately 260 CFU (or approximately 200 LD_50_ equivalents) via subcutaneous injections. Mice were then observed daily for 21 days for clinical signs that would warrant early endpoint euthanasia (see Section 2.4 for clinical scoring details).

### 2.7. Enzyme-Linked Immunosorbent Assay (ELISA)

Immunoglobulin (Ig) class IgG titers (IgG, IgG1, IgG2a, or IgG2c) from vaccinated mice were determined by an ELISA performed in 96-well, Immulon 2 HB, round-bottom plates (ThermoFisher). Irradiated *Y. pestis* CO92 cells (fully virulent encapsulated challenge strain)*, Y. pestis* C12 (non-encapsulated mutant of CO92) cells, or *Y. pestis* CO92 *pgm*^−^/pPst^−^ (VAX) cells were used as antigens diluted in 0.1 M carbonate buffer, pH 9.5, to a concentration of 10 μg/mL. Plates were stored overnight at 4 °C. The plates were washed with washing solution (1× phosphate buffered saline, 0.05% Tween-20), and incubated with blocking solution (1× phosphate buffered saline, 1% casein) for 30 min at 37 °C. Twofold dilutions of mouse sera were made with antibody assay diluent (1× phosphate buffered saline, 0.25% casein) in triplicate, and plates were incubated for 1 h at 37 °C. After the plates were washed, diluted anti-IgG, -IgG1, -IgG2a, or -IgG2c horseradish peroxidase conjugate (1:5000; obtained from Southern Biotechnology Associates, Inc. Birmingham, AL) was added to each well, and plates were incubated for 30 min at 37 °C. After the plates were washed, buffered hydrogen peroxide and 3,3′,5,5′-tetramethylbenzidine solution (Pierce, ThermoFisher) was added to each well, and plates were incubated for 20 min at 37 °C. The reaction was stopped with 2 N sulfuric acid, and the amount of bound antibody was determined colorimetrically by reading at 450 nm with a reference filter (570 nm). The results are reported as the reciprocal of the highest dilution giving a mean OD of at least 0.1 (which was at least twice the background) ± 1 SD.

### 2.8. Spleen Cell Preparations

Splenocytes were collected in accordance with a previously published protocol [60]. Briefly, spleens were excised from euthanized mice (*n* = 5 mice per group), weighed, and disaggregated in Roswell Park Memorial Institute (RPMI) 1640 medium (ThermoFisher, Grand Island, NY). Red cells in the spleen homogenate were lysed with ammonium–chloride–potassium (ACK) Lysing Buffer (BioWhittaker, Walkersville, MD) after the extract was diluted with RPMI 1640 medium and cells pelleted by centrifugation at 335× *g* for 10 min. Splenocytes were then washed once and re-suspended in CTL-Medium supplemented with 1% l-glutamine and the cells counted.

### 2.9. Luminex Cytokine Assay

Cytokine and chemokine expression profiles were measured by the Luminex MagPix (ThermoFisher, Grand Island, NY). Cultured splenocytes were re-stimulated with irradiated *Y. pestis* CO92 (encapsulated fully virulent challenge strain) or *Y. pestis* CO92 *pgm^−^/*pPst*^−^*(VAX) cells (5 μg/mL) in complete medium containing 10% heat-inactivated fetal calf serum (ThermoFisher), 1 mM sodium pyruvate, 0.1 mM non-essential amino acids, 100 U/mL penicillin, 100 μg/mL streptomycin, and 50 μM 2-mercaptoethanol. The splenocytes from each mouse were processed per the Mouse Cytokine Magnetic 36-plex protocol (ThermoFisher). Only the cytokines/chemokines that showed at least a two-fold change relative to the PBS/mock vaccinated group during the study were reported.

### 2.10. ELISpot

The ELISpot assay was performed by seeding purified splenocytes in the presence of irradiated *Y. pestis* CO92 cells, *Y. pestis* C12 cells, or *Y. pestis* CO92 *pgm*^−^/pPst^−^ (VAX) cells. Each mouse was run in duplicate in each of five independent stimulation conditions. A solution of phorbol 12-myristate 13-acetate (PMA; 100 ng/mL) and ionomycin (0.5 μg/mL) was used as the positive control stimulant and resulted in uniformly strong signals (data not shown). Briefly, 96-well plates were coated overnight at 4 °C with 80 μL/well capture anti-mouse interferon-gamma (IFN-γ) monoclonal antibody. Plates were washed one time with 1× phosphate buffered saline. Irradiated *Y. pestis* CO92 cells, *Y. pestis* C12 cells, or *Y. pestis* VAX cells (5 µg/well) were re-suspended in CTL-Medium supplemented with 1% l-glutamine, and 100 µL was added to each well. The plates were incubated at 37 °C, 9% CO_2_ for 15 min. Splenocytes were re-suspended in CTL-Medium supplemented with 1% l-glutamine and seeded at 10^4^ cells per well. Plates were incubated for 24 h at 37 °C, 9% CO_2_; then, splenocytes were removed, and plates were washed twice with phosphate buffered saline alone and then twice with phosphate buffered saline and 0.05% Tween. Next, 80 μL/well biotinylated detection anti-mouse IFN-γ monoclonal antibody was added. After 2 h of incubation at room temperature, plates were washed three times with phosphate buffered saline and 0.05 % Tween. Then, 80 μL of Streptavidin-alkaline phosphatase conjugate antibody solution was added to the wells, and the plates were incubated for 30 min at room temperature. Development reagents were added and incubated for 15 min at room temperature according to manufacturer’s recommendations. The colorimetric reaction was stopped by washing the plates three times with distilled water and air-drying overnight. Spots were scanned and analyzed using an automated ELISpot reader (CTL-Immunospot S6 Analyzer, CTL, Germany). The T-cell response was assessed as spot-forming cells (SFC), adjusted to 10^6^ cells per well, which was automatically calculated by the ImmunoSpot^®^ software for each stimulation condition and the medium-only control.

### 2.11. Pathology

Post-mortem tissues were collected from representative mice and fixed in 10% neutral buffered formalin. Samples were embedded in paraffin and sectioned for hematoxylin and eosin (H&E) staining, as previously described [61]. Histologically, an apoptotic cell appears as a round, shrunken cell with a condensed cytoplasm. Macrophages that engulf these cells are called tingible body macrophages. Immunohistochemistry using anti-F1^+^ *Y. pestis* rabbit polyclonal antibodies (USAMRIID, Frederick, MD) was also performed on select tissues.

### 2.12. Statistical Analyses

Lymph node data were analyzed by both chi-square test and Fisher’s exact test. Titers and ELISpot results were log-transformed prior to analysis, with results summarized as geometric mean and geometric standard error. The log-transformed values were analyzed under a repeated-measures ANOVA model, and the *p*-values for selected post hoc comparisons were reported. Cytokine values were also log-transformed for analysis and, for each stimulation condition, a one-way ANOVA was applied, with post hoc comparisons to PBS control being reported. Animal weights and per capita food and water consumption were analyzed by repeated-measures ANOVA as well, but were not log-transformed prior to analysis. Analyses were performed using Statistical Analysis Software (SAS^®^) version 9.4 (Cary, North Carolina, USA), SAS PROC MIXED procedure.

## 3. Results

### 3.1. Impact of Analgesic Administration on the Clinical Course of the Live Attenuated Y. pestis Vaccine

#### 3.1.1. Clinical Observations

The experimental vaccine used in these experiments was an attenuated mutant of wild-type *Y. pestis* CO92. The deletion mutation of the *pgm* locus and curing of the pPst plasmid resulted in significant attenuation [52,53]. The *pgm* locus encodes a pathogenicity island responsible for iron scavenging and utilization [54,62,63,64]. The pPst plasmid encodes plasminogen activator, a protein critical for lethal infection [53,65,66]. The wild-type CO92 subcutaneous LD_50_ for BALB/c and C57BL/6 mice is estimated to be <2 CFU, whereas the subcutaneous LD_50_ for the *Y. pestis pgm*^−^/pPst^−^ (VAX) strain is estimated to be greater than 1 × 10^8^ CFU [52]. Due to the early nature of the vaccination work described here, we chose to characterize a high vaccination dose. Although the VAX strain was not previously associated with lethality with doses of approximately 10^8^ CFU, the potential exists that infection with any live vaccine could be lethal for rare individuals. Accordingly, this vaccine model offered several important parameters that could be used to evaluate the impact of analgesics on the immune response after vaccination but also in context of a live, potentially lethal, infection model.

Recognizing pain or illness in mice can be difficult because they are prey animals housed in groups, and do not openly display signs of illness or injury until it is severe [31,57]. A clinical observation score sheet was utilized that was designed to allow uniform, early endpoint euthanasia after subcutaneous infection with the live attenuated *Y. pestis* vaccine strain. The average clinical scores of the BALB/c mice (Table 1) demonstrated no statistically significant benefit, based upon clinical scores, attributable to analgesic treatment during the vaccine regimen. The C57BL/6 mice in our study were never observed to have clinical signs, despite documented weight loss. Wilson and colleagues demonstrated that C57BL/6 were highly sensitive to ACE, while BALB/c appeared to be insensitive during antinociception assessment using the writhing test [67]. Thus, variability in pain tolerance, drug sensitivity, and potential immuno-reactogenicity between strains of mice could explain the differences in clinical presentations after vaccination in our study.

#### 3.1.2. Draining Lymph Nodes

Lymph node swelling is a common response to foreign antigen material such as vaccines. Antigen-presenting cells (APCs) are known to enter the lymphatic capillaries and migrate through the vessels to elicit acquired immune responses in the body [68,69]. Enlarged axillary lymph nodes were readily identified during physical examination by a veterinarian in some of the mice starting as early as four days post vaccination and generally lasting through day 11. We observed swelling in 35% of C57BL/6 mice that received VAX or 50% of C57BL/6 mice that received VAX + ACE. Most of the swelling was observed in a single front axillary area of the mouse; however, one mouse receiving VAX and three mice receiving VAX + ACE had appreciable front bilateral axillary lymphadenopathy. There was a single BALB/c mouse outlier which exhibited enlarged axillary lymph nodes after receiving VAX + MEL. The swelling observed in the C57BL/6 mice was likely due to their differential responsiveness to the VAX antigens compared to that of the BALB/c mice [28,70,71]. This differential responsiveness is likely attributable to the T helper 1 (Th1)- or Th2-biased cellular responses in C57BL/6 and BALB/c mice, respectively. The ACE did not appear to limit the physical manifestations of the immune reactivity in the C57BL/6 mice, but rather slightly exacerbated this clinical sign associated with the vaccine. The regulatory T cell (Treg) response suppression that leads to increased lymphadenopathy is one hypothesis which was documented in prior studies [72,73,74]. This lymphadenopathy could be attributable to reduced thymic Treg development or poor maturation in the peripheral circulation that results in greater susceptibility to Treg clearance.

#### 3.1.3. Lethality Associated with Vaccination and Analgesic Treatment

While this vaccine strain is significantly attenuated, the resulting infection can result in low levels of lethality at the dose used in this report. Unexpectedly, there were five mice (four found dead despite multiple checks and one euthanized) that did not survive the vaccination on study day 3. Of these five mice (three C57BL/6 and two BALB/c), all received VAX + MEL. Other BALB/c deaths were noted on days 7–8 (one mouse VAX + ACE on day 7 and three mice receiving VAX alone on day 8). There was a single C57BL/6 (VAX only) mouse death on day 18. When combining these data from both strains of mice, these deaths associated with the vaccine and treatment can be summarized as follows: 12.5% of animals receiving VAX + MEL succumbed on day 3, 2.5% of the animals receiving VAX + ACE succumbed on day 7, and 10% of the animals receiving VAX alone succumbed between days 8 and 18.

**Figure 1:**
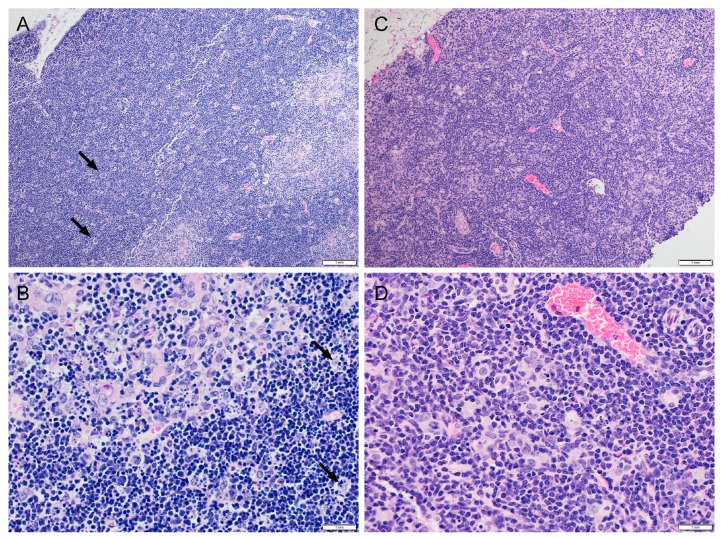
Multifocal lymphocyte apoptosis in the thymus of vaccinated BALB/c mice treated with meloxicam. Data are representative of all mice examined that succumbed after receiving vaccine (VAX) + meloxicam (MEL). (**A**) Thymus, cortex: numerous tingible body macrophages (macrophages that contain cytoplasmic apoptotic lymphocytes) (arrows), female BALB/c mouse, hematoxylin and eosin (H&E) stain, 10×. (**B**) Thymus: multifocal moderate to marked lymphocyte apoptosis, tingible body macrophages (arrows), female BALB/c, H&E stain, 40×. (**C**) Thymus: normal, female BALB/c, H&E stain, 10×; note: not an age-matched control exposed to the same treatment regimen as protocol mice. (**D**) Thymus: normal, female BALB/c, H&E stain, 40×; note: not an age-matched control exposed to the same treatment regimen as protocol mice.

The VAX + MEL-treated mice that succumbed to vaccination were necropsied, and samples were subjected to histopathological analyses. There was no definitive cause of death but the major pathological finding was lymphocyte apoptosis in the lymphoid organs (e.g., spleen, lymph nodes, and thymus) (Figure 1). These early event deaths were only observed in mice that received VAX + MEL. Mice receiving only MEL showed no clinical signs. In mice, lymphocyte apoptosis is often associated with an increase of corticosteroid which was reported to be induced by a wide range of stressors including dehydration, hypothermia, or acute infections [75]. One mouse exhibited degeneration/necrosis of the renal tubular epithelium, which may be related to administration of MEL or a secondary ischemic change. Samples were tested with IFA using anti-encapsulated *Y. pestis* antibodies, and bacteria were not readily identified in the examined tissues.

Reported side effects of NSAIDs are due mostly to their inhibition of COX-1 enzymes and include gastrointestinal ulceration, platelet inhibition, and renal toxicity [76]. MEL, however, preferentially inhibits the COX-2 enzymes which are associated with pain and inflammation. Therefore, the side effects are not normally as prevalent with the use of meloxicam as with COX-1 inhibiting NSAIDs. The five mice examined showed no signs of gastrointestinal ulceration.

#### 3.1.4. Mouse Weights Post Vaccination

**Figure 2:**
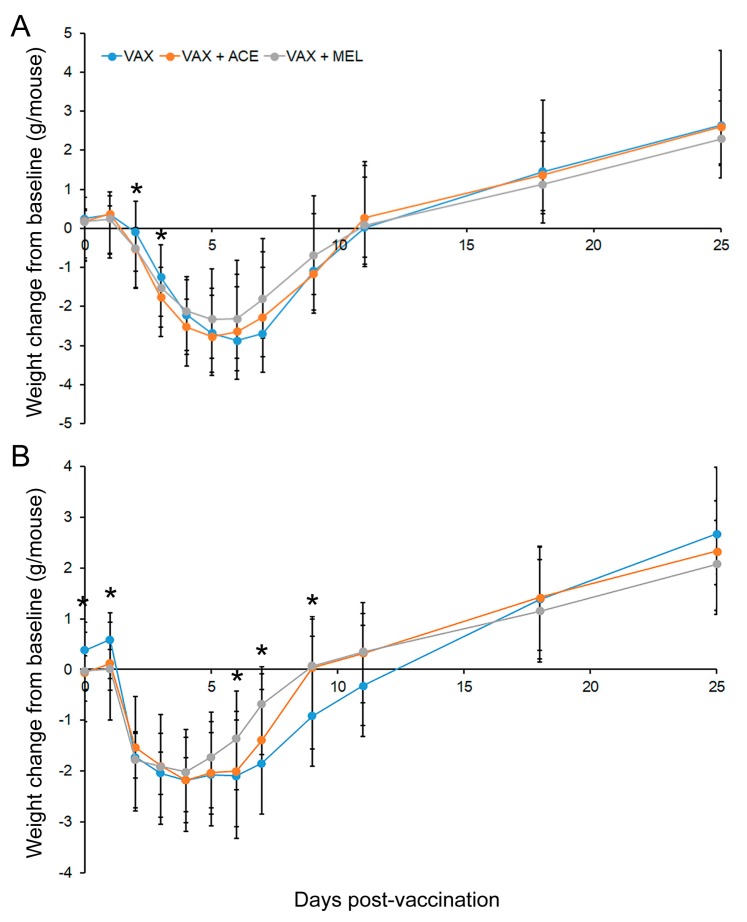
Mouse weights post vaccination. Vaccinated BALB/c and C57BL/6 mice experience significant weight fluctuations even in the presence of analgesia. Change in weight from baseline in (**A**) BALB/c mice and (**B**) C57BL/6 mice through day 25 post vaccination ± analgesic administration. * indicates *p* < 0.05.

Mice were individually weighed daily for nine consecutive days. The weights before analgesic administration on day −1 were used as the baseline weights. Analyses of these actual baseline weights demonstrated that the differences between cages were not statistically significant prior to treatment initiation (*p* > 0.5); starting on day 0, subsequent weights are presented as change from baseline weight. Figure 2 (Tables S1 and S2, Supplementary Materials) depict this change in baseline (day −1) from day of analgesia initiation (day 0) and vaccination (day 1) to day 46. This live attenuated vaccine resulted in significant weight loss starting as early as 24 h post vaccination in both BALB/c and C57BL/6 mice. The weight loss continued, ultimately peaking between study days 4 and 6, at which time the mice began to improve and gain weight returning to approximately baseline on day 11.

In the case of BALB/c mice (Figure 2A), the decrease in weight from baseline was significantly greater in mice that received VAX + ACE or VAX + MEL (*p* < 0.05) on day 2 and greater in mice that received VAX + ACE on day 3 (*p* < 0.05) compared to VAX alone. C57BL/6 mice (Figure 2B) showed significant differences on day 0 and on day 1 for mice that received VAX + ACE or VAX + MEL. While, on average, mice continued to gain weight and were not yet appreciably affected by the infection with the live attenuated vaccine, the extent of weight gain was significantly decreased in mice receiving either VAX + ACE or VAX + MEL. C57BL/6 mice receiving VAX + MEL either lost less weight or began to gain weight at a significantly greater rate than VAX alone mice on days 6, 7, and 9 (*p* < 0.05), whereas C57BL/6 mice receiving VAX + ACE were observed to gain significantly more weight than mice receiving VAX alone only on day 9. It was shown that C57BL/6 mice have an increased sensitivity to acetaminophen compared to BALB/c mice, and this weight gain might suggest reduced distress in the C57BL/6 mice at those time points [67]. When comparing mouse strains, C57BL/6 mice exhibited trends of reduced weight loss that appear to begin and end more rapidly than in BALB/c mice. There were no significant examples of weight loss in non-vaccinated mice only receiving analgesics.

#### 3.1.5. Food and Water Consumption

**Figure 3:**
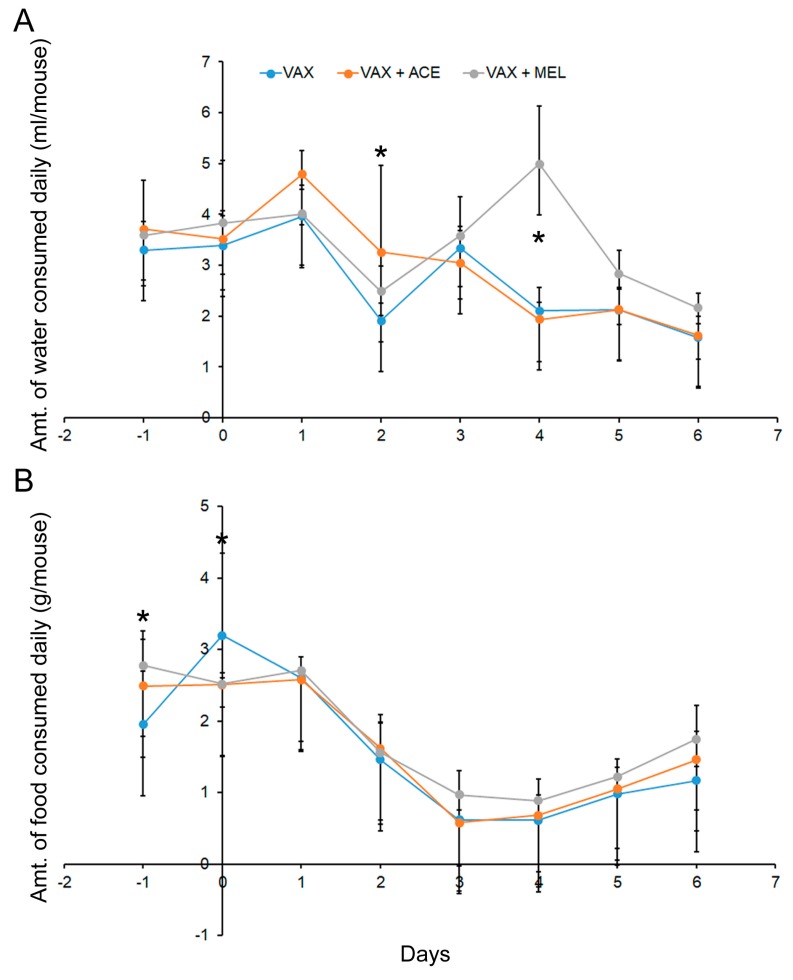
Food and water consumption rates. In order to achieve greater statistical power, the water and food consumption data were combined for both BALB/c and C57BL/6 mice receiving the same vaccine and treatment regimens. (**A**) Water consumption remained comparable amongst all groups with two exceptions. Mice receiving VAX + ACE consumed significantly more water than VAX alone mice on day 2 (*p* < 0.05), and mice receiving VAX + MEL consumed significantly more water than VAX alone mice on day 4 (*p* < 0.05). (**B**) Food consumption also remained comparable amongst all groups and no substantial benefits could be attributed to vaccination or analgesia. * indicates *p* < 0.05.

To evaluate food and water consumption rates, weights of food and water consumed were recorded daily starting on day −2 and ending on day 6 for a total of nine days, and used to calculate the per capita consumption in each pan. Data from C57BL/6 and BALB/c mice were combined for the analysis of food and water consumption in order to increase statistical power (Figure 3 and Table S3, Supplementary Materials). Analyses of food and water consumption indicated that the differences between the strains of mice were not widespread, and these data are depicted in Table S4 and S5 (Supplementary Materials).

On day 2, there was a significant difference (*p* < 0.05) between the VAX + ACE group and the VAX alone group for water consumption (Figure 3 and Table S3, Supplementary Materials). On day 3, there was no significance difference between any of the groups, and consumption continued to decline post vaccination. The VAX + MEL group had a significant increase in water consumption on day 4 (*p* < 0.05). The VAX + MEL group is comparable to the other groups again on day 5; therefore, one explanation of this observation on day 4 is water bottle leakage, and could be an experimental artefact. However, because of the unanticipated deaths of VAX + MEL-treated mice on day 3, the significance of this appreciable spike in water consumption on day 4 cannot be completely discounted.

Combining C57BL/6 and BALB/c mice, the analyses of food consumption revealed very few differences that achieved statistical significance (Figure 3). On day −1, differences in food consumption were noted, but treatment was yet to be initiated at time of data collection. On day 0, however, mice that received either analgesic demonstrated lower consumption rates (*p* < 0.05). The food consumption gradually decreased from day of vaccination to day 3. Starting on approximately day 5, there was a gradual increase, suggesting that the animals were recovering from the effects of the vaccination.

### 3.2. Impact of Analgesic Administration on the Resulting Immune Response after Vaccination with Live Y. pestis Vaccine: Cellular Immune Response

#### 3.2.1. Analgesic Treatment of Mice during Vaccination with VAX Reduced Splenocyte IFN-γ Recall Response

**Figure 4:**
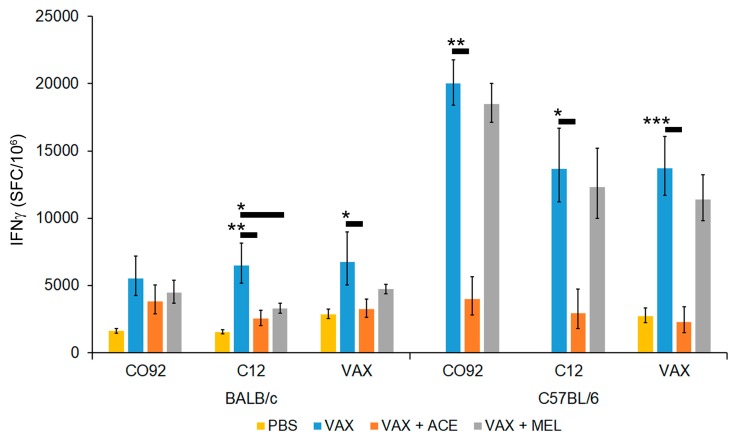
Interferon-gamma (IFN-γ) recall response in vaccinated mice in the presence of analgesia. C57BL/6 or BALB/c mice were vaccinated as described previously. Splenocytes were stimulated in the presence of medium/mock, CO92 cells, C12 cells, or VAX cells (5 µg/mL) for 24 hours at 37 °C. The number of discreet IFN-γ-secreting cells on the membrane was measured as spot-forming cells (SFC) per 10^6^ cells. Data are presented as geometric mean of number of spots per well, and standard error of the mean is also depicted. The *p*-values reflect the result of post hoc comparisons under a repeated-measures ANOVA model. Comparison to VAX group was made by Welch’s *t*-test. * *p* < 0.05, ** *p* < 0.01, *** *p* < 0.0001.

To assess the impact of analgesic treatment that is concurrent with vaccine administration, we vaccinated mice with approximately 10^7^ CFU of *Y. pestis* VAX in the presence or absence of ACE or MEL treatment. At 28 days post vaccination, mice were euthanized and spleens were harvested. Cell specific recall response of splenocytes was assessed by IFN-γ ELISpot assay after re-stimulation with inactivated *Y. pestis* CO92, *Y. pestis* C12, or *Y. pestis* VAX cells. The F1 capsular antigen of *Y. pestis* is immunodominant [77,78,79,80]; therefore, we used the non-encapsulated C12 mutant [51] to assess the immuno-stimulatory potential of non-capsular *Y. pestis* antigens. All groups analyzed contained five mice, except for the C57BL/6 VAX + ACE group which had a single mouse removed from further analysis based on outlier characteristics (Figure S1, Supplementary Materials). Both mouse strains (BALB/c or C57BL/6) vaccinated with *Y. pestis* VAX induced a strong IFN-γ response with all three antigens used for stimulation. The overall induction of IFN-γ was observed to be much greater in C57BL/6 mice compared to BALB/c mice (Figure 4 and Table S6, Supplementary Materials).

Mice that were vaccinated in the presence of either ACE or MEL treatment exhibited an apparent reduced IFN-γ response, although generally statistical significance was only reached in the presence of ACE. Furthermore, there was a greater reduction in IFN-γ in both strains of mice treated with ACE relative to MEL, and this blunted response was more pronounced in C57BL/6 mice (Figure 4 and Table S6, Supplementary Materials).

Stimulation of splenocytes with irradiated CO92 cells promoted a stronger IFN-γ response compared to splenocytes stimulated with C12 cells, or VAX cells in C57BL/6 mice for all vaccinated groups, although these differences failed to reach significance. In contrast, when comparing stimulation of BALB/c splenocytes with CO92 cells to splenocytes stimulated with C12 cells, or the *Y. pestis* VAX cells, the IFN-γ levels were only reduced in splenocytes collected from vaccinated mice that were treated with analgesics and stimulated with C12 cells, but the reduction did not reach statistical significance (Figure 4 and Table S6, Supplementary Materials). Based on these results, there is evidence of an alteration of the IFN-γ-mediated immune response associated with analgesic treatment. The results indicate that ACE has the most profound effect, with the observed immune suppression being dependent on drug and/or mouse strain.

#### 3.2.2. Cytokine Profiles from Vaccinated Mice Differ from Those Obtained from Controls and Show Trends of Cytokine Perturbation Associated with Analgesic Treatment

**Figure 5:**
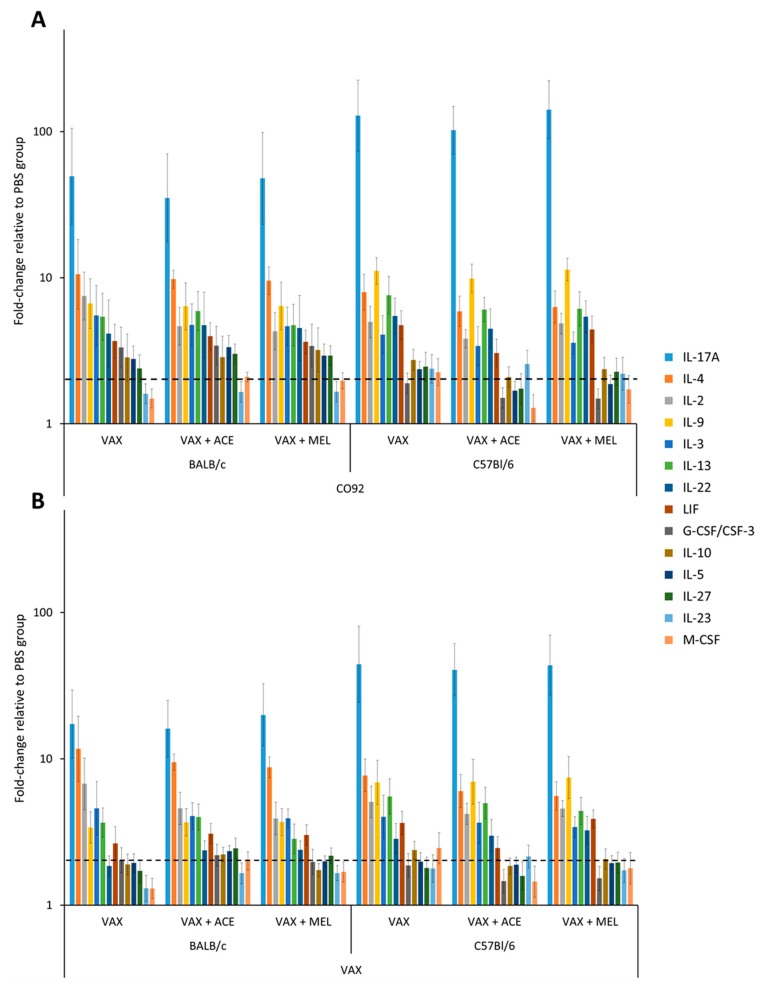
Cytokine response in mice vaccinated with VAX and treated with ACE or MEL. C57BL/6 and BALB/c mice were vaccinated with potassium phosphate buffered solution (PBS) (*n* = 5), VAX (*n* = 5), VAX + ACE (BALB/c *n* = 5, C57BL/6 *n* = 4), or VAX + MEL (*n* = 5). At day 21 post vaccination, mice were euthanized and spleens were harvested. Splenocytes (10^6^ cells) were re-stimulated for 48 h in the presence of (**A**) irradiated CO92 cells (5 μg/mL) or (**B**) irradiated VAX cells (5 µg/mL). Dashed line denotes a two-fold increase over PBS/mock vaccinated control group. The levels of cytokines were measured by the Luminex bead-based suspension immunoassay. Graph values are expressed as a fold-change compared to PBS group for each analyte. Values depicted on the graph are expressed as fold-change compared to PBS groups and standard error of the mean for each analyte is also depicted.

In order to further characterize and compare the cytokine response produced by vaccinated C57BL/6 and BALB/c mice, harvested splenocytes were stimulated with irradiated CO92 cells or *Y. pestis* VAX cells, and supernatants were analyzed by bead-based suspension immunoassay. In both strains of mice, the vaccination upregulated a multitude of cytokines upon stimulation with CO92 cells, with the highest fold-change difference relative to the PBS/mock vaccinated group observed in interleukin- 17A (IL-17A), IL-4, IL-2, IL-9, IL-3, IL-13, IL-22, leukemia inhibitory factor (LIF), IL-27, and IL-10 (Figure 5; Tables S7 and S8, Supplementary Materials). Splenocytes from C57BL/6 mice exhibited a higher overall IL-17A, IL-9, IL-13, and IL-23 response relative to splenocytes from BALB/c mice upon stimulation with CO92 cells, while splenocytes from BALB/c mice induced a higher overall IL-4, IL-2, IL-3, Granulocyte colony-stimulating factor (G-CSF), IL-10, IL-5, and IL-27 response relative to C57BL/6 mice upon stimulation with CO92 cells (Tables S7 and S8, Supplementary Materials). With the exception of IL-10 and IL-5, the cytokine pattern remained similar upon stimulation with *Y. pestis* CO92 or *Y. pestis* VAX cells for both strains of mice (Figure 5). The most overt difference between stimulation conditions was the magnitude of the overall cytokine response that was markedly greater with *Y. pestis* CO92 than cytokines induced by the attenuated VAX strain.

Upon addition of analgesic treatment to the vaccine regimen, there was an appreciable reduction in the expression of IL-17A in both strains of mice treated with ACE (Tables S7 and S8, Supplementary Materials). On the other hand, in the presence of MEL, the level of IL-17A remained comparable to VAX only treatment group. The level of IL-2 was depressed in BALB/c mice in the presence of both analgesics, with greater inhibition in the presence of MEL. Contrary to this, in C57BL/6 mice, ACE treatment reduced IL-2 levels to a greater extent than MEL treatment. Furthermore, the expression levels of IL-3, and IL-4 in both strains of mice were also reduced in the presence of both analgesic treatments. Splenocytes re-stimulated with CO92 from VAX + ACE-treated C57BL/6 mice had reduced expression of IL-9, IL-13, IL-22, LIF, IL-10, IL-5, IL-27, G-CSF/CSF-3, and Macrophage colony-stimulating factor (M-CSF). BALB/c mice exhibited a slight enhancement in IL-5, IL-13, IL-27, LIF, and M-CSF in the presence of ACE treatment. While the impacts of analgesia on cytokine production were not statistically significant, even subtle differences could be biologically relevant and contribute to immune modulation.

### 3.3. Impact of Analgesic Administration on the Resulting Immune Response after Vaccination with Live Y. pestis Vaccine: Humoral Immune Response

#### 3.3.1. Total IgG Titers Were Not Significantly Impacted by Analgesia at Time of Vaccination

Sera collected 28 days post-vaccination were analyzed by ELISA to determine total IgG titers against either irradiated *Y. pestis* VAX cells or *Y. pestis* CO92 cells. While there were trends of decreased antibody production associated with analgesic administration, the comparisons of geometric means of the titers did not reach statistical significance (Table S9, Supplementary Materials).

#### 3.3.2. The IgG2a-to-IgG1 Titer Ratios Were Significantly Altered in BALB/C by the Use of Acetaminophen or Meloxicam at the Time of Vaccination

**Figure 6:**
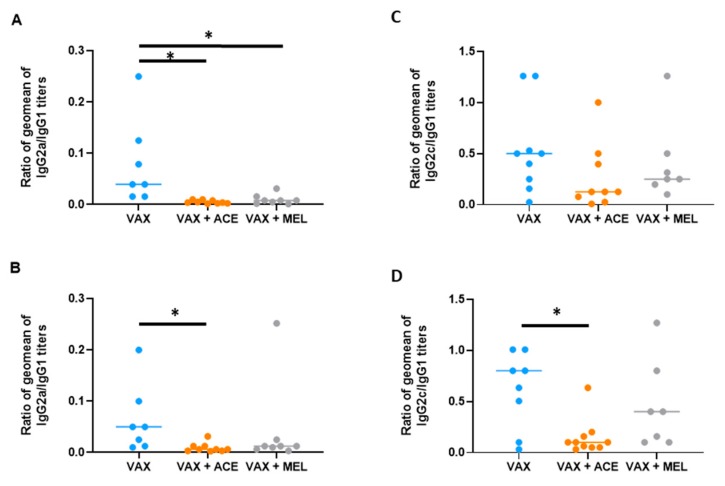
Immunoglobulin (Ig)G2a/IgG1 and IgG2c/IgG1 titer ratios in the presence of analgesia. IgG2a/IgG2c response is suppressed in vaccinated BALB/c and C57BL/6 mice. Sera were analyzed by ELISA using irradiated VAX cells or irradiated CO92 cells as capture antigen at a concentration of 10 μg/mL. The ratios of IgG2a/IgG1 antibody titers was calculated from sera of (**A**) BALB/c mice against irradiated VAX cells; VAX compared to VAX + ACE, *p* = 0.001; VAX compared to VAX + MEL, *p* = 0.015; (**B**) BALB/c mice against irradiated CO92 cells; VAX compared to VAX + ACE, *p* = 0.013; (**C**) C57BL/6 mice against irradiated VAX cells. For C57BL/6 VAX + ACE mice anti-VAX titers, one mouse demonstrated an IgG2c/IgG1 ratio of 2.52; this mouse was included for statistical analyses but excluded for graphical representation. (**D**) C57BL/6 mice against irradiated CO92 cells; VAX compared to VAX + ACE, *p* = 0.021. For C57BL/6 VAX mice anti-CO92 titers, one mouse demonstrated an IgG2c/IgG1 ratio of 4.03; this mouse was included for statistical analyses but excluded for graphical representation. * denotes *p* < 0.05.

Sera were analyzed by ELISA to determine the individual titers of IgG subclasses IgG2a and IgG1 which are indicative of a Th1- or Th2-biased response, respectively. The ratio of IgG2a to IgG1 may reveal the extent of immune response polarization generated by the vaccine strategy being evaluated. There were significant differences (*p* < 0.01) when comparing this ratio in BALB/c mice receiving VAX alone or in combination with analgesia (Figure 6A,B; Table S10, Supplementary Materials). Both ACE and MEL caused a statistically significant decrease in this ratio, suggesting that the presence of analgesia skewed the immune response to reflect more of a Th2-biased response compared to vaccinated animals receiving no analgesic.

#### 3.3.3. The IgG2c-to-IgG1 Titer Ratios Were Significantly Altered in C57BL/6 Mice by the Use of Acetaminophen at the Time of Vaccination

Sera were analyzed by ELISA to determine the individual titers of IgG subclasses IgG2c for C57BL/6 mice. It was previously demonstrated that C57BL/mice produce IgG2c as opposed to IgG2a [81,82]. The IgG2c and IgG1 ratio is also indicative of a Th1- or Th2-biased polarization. There were no significant differences (*p* > 0.05) when comparing this ratio in MEL-treated vaccinated C57BL/6 for either the VAX or CO92 ELISA antigens. However, ACE treatment resulted in IgG2c suppression and reduction in the IgG2c ratio against either VAX or CO92 ELISA antigens, although statistical significance was only reached for the CO92 ELISA antigen (*p* = 0.021) (Figure 6C,D; Table S10, Supplementary Materials).

### 3.4. While Analgesia Did Alter Several Parameters of the Immune Response to the Vaccination, the Mice Were Protected from Infection with Virulent Y. pestis

Twenty-eight days (study day 29) post vaccination, BALB/c mice were challenged with approximately 130 LD_50_ equivalents and C57BL/6 mice were challenged with approximately 200 LD_50_ equivalents of virulent *Y. pestis* CO92. All control animals succumbed or were euthanized after meeting early endpoint euthanasia criteria by day 7 post challenge. All vaccinated mice, regardless of analgesic treatment, survived the ensuing bacterial challenge and demonstrated no signs of disease.

#### 3.4.1. Total IgG Titers of Mice Surviving Bubonic Plague Challenge Were Not Significantly Impacted by Analgesia Administered at Time of Vaccination

Total IgG was determined by ELISA in sera collected from surviving mice 21 days post challenge with fully virulent *Y. pestis* CO92. Irradiated VAX cells were used as capture antigen to measure any VAX specific booster response associated with the virulent challenge organism. Sera collected from survivors demonstrated that the infection with the fully virulent challenge strain *Y. pestis* CO92 resulted in relatively comparable boosting in the mice, regardless if analgesics were administered during the vaccination (Table S11, Supplementary Materials).

#### 3.4.2. Mouse Weights Post Challenge

**Figure 7:**
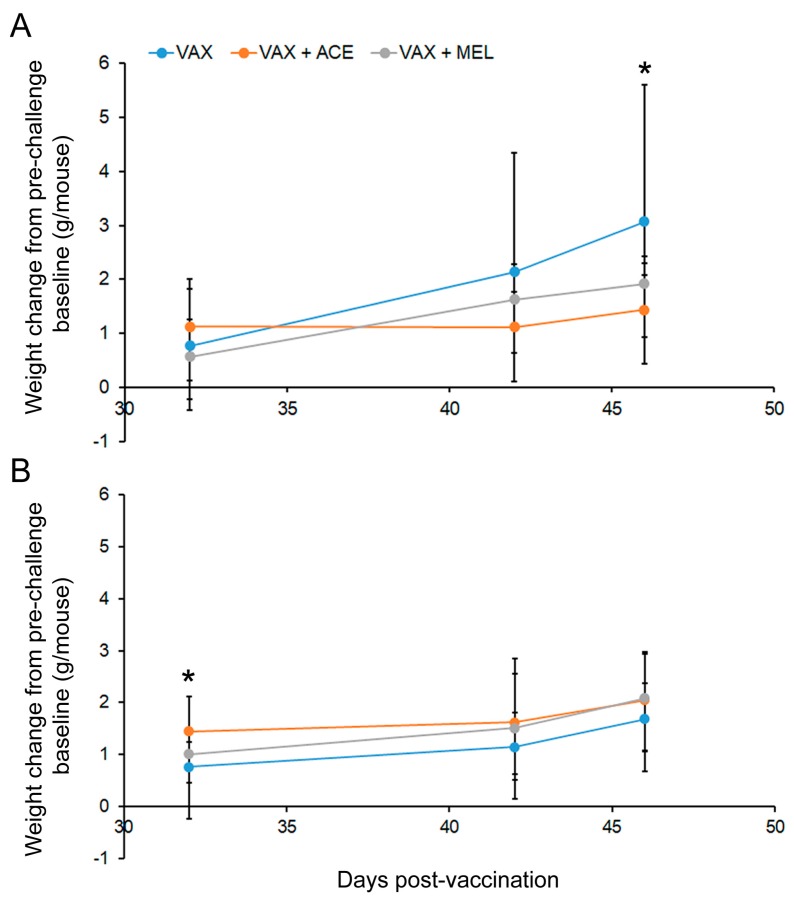
Mouse weights post challenge. Vaccinated BALB/c mice in the presence of analgesia gained less weight after surviving the bubonic plague challenge. Change in weight from baseline in (**A**) BALB/c mice and (**B**) C57BL/6 mice through day 46 post vaccination ± analgesia administration. * indicates *p* < 0.05.

After challenge with fully virulent *Y. pestis* CO92 under ABSL-3 conditions 28 days post vaccination, weights were collected on day 32, day 42, and day 46. The weights were then compared to day-25 weights (pre-challenge baseline weights). As demonstrated in Figure 7 (Tables S1 and S2, Supplementary Materials, post-challenge weights), BALB/c mice that were vaccinated but did not receive analgesia gained significantly more weight after surviving the bubonic plague challenge by day 46 as compared to mice that received ACE during the vaccination regime (*p* < 0.05). C57BL/6 mice that received VAX + ACE did weigh significantly more at day 32. However, C57BL/6 mice showed no differences associated with weight change compared to baseline after surviving the bubonic plague challenge model by day 46 (Figure 7B).

## 4. Discussion 

The use of analgesics is a constantly debated aspect of laboratory animal research and medicine. Literature exists that supports the addition of analgesia; however, there are also data that demonstrate the potential risk associated with its use, particularly when performing early discovery-oriented research. Our data described here suggest that even limited duration of either meloxicam or acetaminophen treatment significantly alters the natural immune response to a live attenuated bacterial vaccine, and neither offer appreciable benefit to the mice in terms of reducing pain or distress associated with vaccination as measured by the parameters of clinical score, weight loss, or food/water consumption. Our study examined a single inoculation with an experimental live *Y. pestis* vaccine with only limited duration analgesia administration. We did not examine the impact of these analgesics on subsequent booster vaccinations. We did, however, examine the antibody titers of the animals that survived bubonic plague. Because the protective immunity associated with this vaccine is substantial, the challenge dose with virulent *Y. pestis* resulted in a sub-clinical infection which inherently acts as booster, at least for antigens that are expressed in both the vaccine strain and the virulent challenge strain.

For our experiments, we chose to examine the impacts of ad libitum acetaminophen on our live experimental attenuated *Y. pestis* vaccine candidate. Meloxicam was chosen because it is frequently used for its antipyretic, anti-inflammatory, antiexudative, and analgesic properties [83,84,85]. While acetaminophen is frequently administered in the drinking water of rodents [27,86], meloxicam is given via parenteral injection. We acknowledge that ad libitum administration may not be reflective of how human patients self-medicate, but there are many advantages to self-administration of medications in a laboratory setting including reduced stress for the animals and no injection site pain. Administering meloxicam by injection to experimental animals is as invasive as most vaccinations. While, in our case, live vaccines resulting in a subclinical infection may be particularly painful and different routes of administration can cause differing levels of pain, giving an injectable analgesic as pain relief to prevent distress from another injection may seem counterintuitive. Additionally, when there are risks of occupational exposure to infectious diseases and concerns of personnel safety in areas of increased biosafety levels (e.g., ABSL-3 or ABSL-4), oral ad libitum is preferred over injections.

Our studies demonstrated several statistically significant differences regarding animal weight and food/water consumption. We measured these parameters, in addition to physical examinations and clinical scoring, to ascertain any benefits attributable to analgesic administration. While we did reach statistical significance at distinct time points, the biological significance of these findings remains in question. It can be surmised based upon these collective data that the analgesia did not demonstrably improve the physical health or wellbeing of the experimental animals being vaccinated. In fact, the early deaths associated with meloxicam paired with vaccination and exacerbated swelling of lymph nodes in C57BL/6 mice receiving acetaminophen may suggest contraindication of such treatment. This point is particularly important in context of associated alterations in the immune response induced by analgesics.

Inbred mouse strains, specifically C57BL/6 and BALB/c, have a distinct propensity to develop biased Th1- or Th2-like immune responses, respectively [87,88,89]. The biased T-cell-mediated immune responses in these strains of mice result in BALB/c mice being susceptible and C57BL/6 mice being resistant to certain intracellular Gram-negative bacteria, such as *Yersinia enterocolitica* [90,91]. The prototypical Th1 cytokine, IFN-γ, was highly expressed in C57BL/6 relative to BALB/c mice after vaccination in both the presence and absence of analgesic treatment. Previous studies also demonstrate a similar mouse strain-dependent IFN-γ profile in mice infected with *Yersinia enterocolitica* [92]. Furthermore, relative to the PBS/mock vaccinated mouse group, there was a higher fold change in IL-4 and IL-5, Th2-associated cytokines, in splenocytes from BALB/c mice relative to C57BL/6 mice stimulated by CO92 cells [93]. In C57BL/6 mice, there was a greater fold change in IL-23 and IL-17 relative to BALB/c mice stimulated with CO92 and the *Y. pestis* VAX. IL-23 is produced by dendritic cells and macrophages upon recognition of bacteria by their pathogen-associated molecular pattern recognition receptors, which in turn promotes the development of Th17 cells. Th17, cluster of differentiation 8 (CD8^+^), and γδ T cells produce IL-17, which enhances T-cell priming, triggers potent inflammatory responses, and stimulates granulopoiesis and extracellular bacterial clearance [94,95].

Vaccinated mice that received analgesic treatment showed depressed recall response of IFN-γ in splenocytes from C57BL/6 and BALB/c mice. The reduction in the IFN-γ response in both mouse strains reached statistical significance in the presence of acetaminophen. Analgesic treatment also reduced the IL-2 response in both strains of mice, but this was more pronounced in BALB/c mice. Disruption in IL-2 levels may, in turn, diminish the growth factor activity of IL-2 on antigen-activated, clonal T-cell expansion and T-regulatory-cell production [96,97]. Also, IFN-γ and IL-2 suppression by way of COX-2 inhibition may promote a selective Th2 environment depending on the tissues and impede elimination of intracellular bacteria [98,99,100,101]. The IL-17 response was reduced in both strains of mice that received acetaminophen, which may predispose them to more protracted pathogen clearance, especially if this results in reduced granulopoiesis [102]. A significant IL-17 response was also documented when looking at samples collected from humans receiving a live plague vaccine [46]. In C57BL/6 mice, reduced granulopoiesis and monopoiesis, due to acetaminophen, may also be further impaired with reduced levels of G-CSF and M-CSF leading to acquired cytopenia [103]. The reduction in IL-10 expression in the presence of acetaminophen treatment in C57BL/6 mice may promote antigen presentation while further hindering the generation of adequate regulatory T-cell response [104,105,106]. The level of leukemia inhibitory factor (LIF) was also reduced in the acetaminophen-treated C57BL/6 mice. Bacterial lipopolysaccharide is able to induce LIF, which plays an anti-inflammatory role in preventing septic shock [107]. LIF may also promote the induction of Foxp3^+^ regulatory cells [108].

Acetaminophen treatment during vaccination may predispose the host to suboptimal immune function, specifically by hindering the induction of Th17-like and Th1-like responses. Also, potential reduction in regulatory T-cell responses may break or by-pass immunological tolerance, leading to higher likelihood of generating self-reactive auto-antibodies and auto-reactive T cells. There is a notable reduction in Treg cells post-surgery in patients undergoing tumor resection with general anesthesia [109,110,111]. Levels of Treg cells are implicated in vaccine immunogenicity [112], as well as the autoimmune sequelae of idiopathic thrombocytopenic purpura, which is correlated with vaccination or infection [113,114,115]. an increase in Th17/Treg ratio may lead to greater inflammation, resulting in enhanced pathogen clearance at the cost of greater susceptibility to bystander immunopathology [116,117,118].

While there were no significant differences in total IgG titers, there were significant differences noted in the ratios of IgG subclasses IgG2a/IgG1 (BALB/c) or IgG2c/IgG1 (C57BL/6) mice (Figure 6). These lower ratios are indicative of either a Th1 or Th2 response, and this was extensively characterized in the case of anti-*Leishmania* vaccine strategies [119,120]. The lower ratios observed in vaccinated BALB/c and C57BL/6 mice that received either acetaminophen or meloxicam suggest that the analgesic treatment induces a more Th2-biased response compared to mice vaccinated without analgesia. Of the two mice strains, the overall Th1-like response was greater in C57BL/6 mice; hence, in the presence of analgesic treatment, the magnitude of IgG2c suppression was more noticeable. Furthermore, acetaminophen treatment caused a greater suppression of IgG2a/c than meloxicam in both strains of mice. Since IFN-γ stimulates B-cell secretion of IgG2a/c, the drastic reduction in the number of IFN-γ-secreting splenocytes in the presence of acetaminophen supports the suppression of antigen-specific IgG2a/c secretion [121,122,123].

These data should also be discussed in the context of administering analgesics during the challenge phase of vaccine studies. There are several examples where the potency of vaccines is increased because the challenge organism results in unapparent or subclinical infections, but ultimately results in a vaccine booster [124,125,126,127]. This was demonstrated in studies with the human anthrax vaccine [125]. The authors demonstrated that challenging the vaccinated animals with fully virulent *Bacillus anthracis* spores resulted in a significant increase in antibody titers to protective antigen (PA) in non-human primates, the major immunogen of the human anthrax vaccine [125]. This was the case when the animals were challenged eight weeks after vaccination or 100 weeks after vaccination. Similar data were demonstrated for humans vaccinated against measles. After exposure to subclinical measles, the antibody titers were appreciably greater [127]. It can be hypothesized that, even in the absence of vaccination, long-term immunity can be maintained with frequent encounters with subclinical or unapparent infections. Thus, our data and those of others suggest that the administration of analgesia during the challenge phase of an experimental vaccine study might mask the true potential of the vaccine being evaluated. If analgesia is attempted at first sign of pain or distress, disease progression and the subsequent immune response could be significantly altered.

It is important to note that we only examined female mice in this current study. Male subjects should be examined during advanced development of novel vaccines, and there are documented differences in vaccine efficacy depending upon the sex of the vaccinee [128,129]. However, the inclusion of male mice results in confounding variables that are problematic during vaccine discovery. In addition to obvious anatomical differences there are also significant differences with hormone levels and social interactions (e.g., aggression). It is well known that male mice are considerably more aggressive than female mice [130,131]. These social interactions often resulted in aggressive behavior and the mice exhibiting signs of increased stress level and/or physical injury [132]. These differences that are observed in group-housed male mice are likely not reflective of higher mammalian disease models. Previous studies demonstrated differences between males and females with regard to pain and analgesia, but these data vary based upon species of animal, pain model used, and type of analgesic administered [130,131,132,133,134,135,136,137,138,139,140,141]. Thus, sex differences can also be confounding variables when examining pain and analgesia as well. Very recently, Bowen et al. demonstrated significant gender-biased survival rates observed in BALB/c and C57BL/6 mice [128]. These authors demonstrated that vaccinated female mice were significantly more likely to survive challenge with virulent *Y. pestis* when compared to their male counterparts. However, the authors could not attribute this gender bias to differences associated with either humoral or cellular vaccine-induced immunity. More work is required to elucidate the reason for these differences in order to ascertain the utility of male mice in early vaccine development.

When performing vaccine discovery using laboratory research animal subjects, additional ethical and legal concerns for animal welfare must be addressed in approved study designs. In vaccine development studies, there are arguments both for and against the use of analgesics. The Guide for the Care and Use of Laboratory Animals mandates that “unless the contrary is established, investigators should consider that procedures that cause pain or distress in human beings may cause pain or distress in other animals” [55]. The Animal and Plant Health Inspection Service [139] currently defines pain as “any procedure that would reasonably be expected to cause more than slight or momentary pain or distress in a human being to which the procedure was applied, that is, pain in excess of that caused by injections or other minor procedures”. Animal welfare rightfully continues to be of the utmost importance, and analgesia could be appropriate during advanced development of infectious disease models and/or vaccination studies, particularly when modeling translational medicine [140,141]. However, these vaccines and animal models must be well-characterized in the absence of analgesics initially, and the analgesics should be shown to offer measurable benefit to the animals with regard to the alleviation of pain or distress. Our *Y. pestis* experimental vaccine, despite immune alterations attributable to analgesic administration, protected all mice in the bubonic plague challenge model. These data presented here explored the impact of analgesic administration on a single dose of a live vaccine. Future work could include examining the impact of analgesia on subunit vaccines and boosting doses (for live vaccines, subunit vaccines, or heterologous strategies), as well as during the challenge phase of the experiment. Additional studies are also required to better develop correlates of immunity associated with plague vaccines in general (both humoral and cellular), and studies are underway to elucidate protection thresholds afforded by this vaccine. Lastly, experiments should be performed to understand the consequences of the diminished IFN-γ response described here, particularly in the context of murine models (to include outbred mouse strains) of plague. These data described in this report range from statistically significant to modest alterations/observations. When analyzed in total, however, changes in these biological parameters have the potential to alter the native immune function in a confounding manner.

## 5. Conclusions

In totality, our immunological data demonstrate a clear alteration of the resulting immune response even when a short duration of analgesics is used during a live vaccine protocol. The mechanisms responsible for this alteration are likely a collection of immune blunting scenarios including altered antigen presentation/processing and cellular recruitment. Different classes of analgesics can have variable effects on the immune response. Opioids are known to alter the immune response in different ways; for example, morphine, fentanyl, and buprenorphine were shown to impact the immune system in distinct ways [142]. Other analgesics such as NSAIDs were shown to modulate cytokine expression, nitric-oxide production, antigen processing/presentation, and cell–cell interactions to include disruption in T-cell proliferation [143,144]. Thus, our data and previously published literature indicate that the impact of analgesia on an immune response is the result of a multi-factorial and complex cascade of events that warrants significant future research efforts.

## Figures and Tables

**Table 1:** Average clinical score per treatment group.

	Clinical Score ^a^ (SD) ^b^		
**Treatment**	Day 0	Day 1	Day 2
**VAX**	2.0 (1.4)	3.0 (1.8)	3.5 (1.7)
**VAX + ACE**	2.0 (2.4)	3.0 (1.8)	2.8 (2.6)
**VAX + MEL**	0.5 (1.0)	3.5 (1.7)	2.0 (1.2)

^a^ Clinical Score parameters evaluated included appearance (i.e. coat appearance, grooming habits, presence of ocular or nasal discharge, posture), natural behavior (i.e. Peer interaction, mobility, alertness, restlessness), and provoked behavior (i.e. reaction to physical stimulation. ^b^ Values depict average clinical score and standard deviation (SD). *N* = 10 mice per pan, 2 pans per group, each pan scored by 2 individuals in a semi-blinded fashioned.

## Supplementary Materials

The following are available online at https://www.mdpi.com/2076-393X/7/4/205/s1: Figure S1: ELISpot assay well images of discrete IFN-γ-secreting splenocytes on the membrane. Stimulation conditions are denoted on the left (medium, *Y. pestis* CO92, *Y. pestis* C12, and *Y. pestis* VAX), mouse vaccine groups are denoted on top (PBS, VAX, VAX + ACE, and VAX + MEL), and mouse strains are denoted on the right (BALB/c and C57BL/6). Arrow denotes an outlier mouse from the C57BL/6 VAX + ACE group that was removed from further analysis; Table S1: Change in weight from baseline in BALB/c mice through day 46 post vaccination ± analgesia administration; Table S2: Change in weight from baseline in C57BL/6 mice through day 46 post vaccination ± analgesia administration; Table S3: Total food and water consumption rates (combined data for BALB/c and C57BL/6; Table S4: Food consumption rates (BALB/c and C57BL/6 mice); Table S5: Water consumption rates (BALB/c and C57BL/6 mice); Table S6: ELISpot IFN-γ responses in VAX-vaccinated BALB/c and C57BL/6 mice treated concurrently with analgesia; Table S7: Comparison of analgesia treatment on the cytokine response in VAX-vaccinated BALB/c mice; Table S8: Comparison of analgesia treatment on the cytokine response in VAX-vaccinated C57BL/6 mice; Table S9: Total IgG titers determined by ELISA in sera collected from mice 28 days post vaccination ± analgesia; Table S10: IgG1 and IgG2a/c titers determined by ELISA in sera collected from mice 28 days post vaccination ± analgesia; Table S11: Total IgG titers determined by ELISA in sera collected from surviving mice 21 days post challenge with fully virulent *Y. pestis* CO92.

## References

[1] Saleh E., Swamy G.K., Moody M.A., Walter E.B. (2017). Parental approach to the prevention and management of fever and pain following childhood immunizations: A survey study. Clin. Pediatr..

[2] Saleh E., Moody M.A., Walter E.B. (2016). Effect of antipyretic analgesics on immune responses to vaccination. Hum. Vaccin. Immunother..

[3] Prymula R., Siegrist C.A., Chlibek R., Zemlickova H., Vackova M., Smetana J., Lommel P., Kaliskova E., Borys D., Schuerman L. (2009). Effect of prophylactic paracetamol administration at time of vaccination on febrile reactions and antibody responses in children: Two open-label, randomised controlled trials. Lancet.

[4] Homme J.H., Fischer P.R. (2010). Randomised controlled trial: Prophylactic paracetamol at the time of infant vaccination reduces the risk of fever but also reduces antibody response. Evid. Based Med..

[5] Prymula R., Habib A., Francois N., Borys D., Schuerman L. (2013). Immunological memory and nasopharyngeal carriage in 4-year-old children previously primed and boosted with 10-valent pneumococcal non-typeable *Haemophilus influenzae* protein D conjugate vaccine (PHiD-CV) with or without concomitant prophylactic paracetamol. Vaccine.

[6] Whaley K., Sloane D.J. (1975). Studies of the action of some anti-inflammatory drugs on complement mediated immune haemolysis. Br. J. Clin. Pharmacol..

[7] Kolstad A.M., Rodriguez R.M., Kim C.J., Hale L.P. (2012). Effect of pain management on immunization efficacy in mice. J. Am. Assoc. Lab. Anim. Sci..

[8] Minor P.D. (2015). Live attenuated vaccines: Historical successes and current challenges. Virology.

[9] Detmer A., Glenting J. (2006). Live bacterial vaccines—A review and identification of potential hazards. Microb. Cell Fact..

[10] Chiu I.M. (2018). Infection, Pain, and Itch. Neurosci. Bull..

[11] Blake K.J., Baral P., Voisin T., Lubkin A., Pinho-Ribeiro F.A., Adams K.L., Roberson D.P., Ma Y.C., Otto M., Woolf C.J. (2018). *Staphylococcus aureus* produces pain through pore-forming toxins and neuronal TRPV1 that is silenced by QX-314. Nat. Commun..

[12] Das R.R., Panigrahi I., Naik S.S. (2014). The effect of prophylactic antipyretic administration on post-vaccination adverse reactions and antibody response in children: A systematic review. PLoS ONE.

[13] Manley J., Taddio A. (2007). Acetaminophen and ibuprofen for prevention of adverse reactions associated with childhood immunization. Ann. Pharmacother..

[14] Fleischmann T., Arras M., Sauer M., Saleh L., Rulicke T., Jirkof P. (2017). Voluntary intake of paracetamol-enriched drinking water and its influence on the success of embryo transfer in mice. Res. Vet. Sci..

[15] Theisen E., McDougal C.E., Nakanishi M., Stevenson D.M., Amador-Noguez D., Rosenberg D.W., Knoll L.J., Sauer J.D. (2018). Cyclooxygenase-1 and -2 play contrasting roles in *Listeria*-stimulated immunity. J. Immunol..

[16] Vinegar R., Truax J.F., Selph J.L. (1976). Quantitative comparison of the analgesic and anti-inflammatory activities of aspirin, phenacetin and acetaminophen in rodents. Eur. J. Pharmacol..

[17] Gladtke E. (1983). Use of antipyretic analgesics in the pediatric patient. Am. J. Med..

[18] Botting R.M. (2000). Mechanism of action of acetaminophen: Is there a cyclooxygenase 3?. Clin. Infect. Dis..

[19] Yaffe S.J. (1981). Comparative efficacy of aspirin and acetaminophen in the reduction of fever in children. Arch. Intern. Med..

[20] Temple A.R. (1983). Review of comparative antipyretic activity in children. Am. J. Med..

[21] Cuestas E. (2010). Acetaminophen may decrease antibody response in infants being immunized. J. Pediatr..

[22] Bancos S., Bernard M.P., Topham D.J., Phipps R.P. (2009). Ibuprofen and other widely used non-steroidal anti-inflammatory drugs inhibit antibody production in human cells. Cell. Immunol..

[23] Chandrasekharan N.V., Dai H., Roos K.L.T., Evanson N.K., Tomsik J., Elton T.S., Simmons D.L. (2002). COX-3, a cyclooxygenase-1 variant inhibited by acetaminophen and other analgesic/antipyretic drugs: Cloning, structure, and expression. Proc. Natl. Acad. Sci. USA.

[24] Simmons D.L., Wagner D., Westover K. (2000). Nonsteroidal anti-inflammatory drugs, acetaminophen, cyclooxygenase 2, and fever. Clin. Infect. Dis..

[25] Cooper D.M., DeLong D., Gillett C.S. (1997). Analgesic efficacy of acetaminophen and buprenorphine administered in the drinking water of rats. Contemp. Top. Lab. Anim. Sci..

[26] Dickinson A.L., Leach M.C., Flecknell P.A. (2009). The analgesic effects of oral paracetamol in two strains of mice undergoing vasectomy. Lab. Anim..

[27] Flecknell P. (2018). Rodent analgesia: Assessment and therapeutics. Vet. J..

[28] Flecknell P.A. (1984). The relief of pain in laboratory animals. Lab. Anim..

[29] Furedi R., Bolcskei K., Szolcsanyi J., Petho G. (2009). Effects of analgesics on the plantar incision-induced drop of the noxious heat threshold measured with an increasing-temperature water bath in the rat. Eur. J. Pharmacol..

[30] Lahoti A., Kalra B.S., Tekur U. (2014). Evaluation of the analgesic and anti-inflammatory activity of fixed dose combination: Non-steroidal anti-inflammatory drugs in experimental animals. Indian J. Dent. Res..

[31] Matsumiya L.C., Sorge R.E., Sotocinal S.G., Tabaka J.M., Wieskopf J.S., Zaloum A., King O.D., Mogil J.S. (2012). Using the Mouse Grimace Scale to reevaluate the efficacy of postoperative analgesics in laboratory mice. J. Am. Assoc. Lab. Anim. Sci..

[32] Mickley G.A., Hoxha Z., Biada J.M., Kenmuir C.L., Bacik S.E. (2006). Acetaminophen self-administered in the drinking water increases the pain threshold of rats (*Rattus norvegicus*). J. Am. Assoc. Lab. Anim. Sci..

[33] Richardson C.A., Flecknell P.A. (2005). Anaesthesia and post-operative analgesia following experimental surgery in laboratory rodents: Are we making progress?. Altern. Lab. Anim..

[34] Srikiatkhachorn A., Tarasub N., Govitrapong P. (1999). Acetaminophen-induced antinociception via central 5-HT(2A) receptors. Neurochem. Int..

[35] St A.S.L., Martin W.J. (2003). Evaluation of postoperative analgesia in a rat model of incisional pain. Contemp. Top. Lab. Anim. Sci..

[36] Liu B., Qu L., Yan S. (2015). Cyclooxygenase-2 promotes tumor growth and suppresses tumor immunity. Cancer Cell Int..

[37] Friton G.M., Schmidt H Fau-Schrodl W., Schrodl W. (2006). Clinical and anti-inflammatory effects of treating endotoxin-challenged pigs with meloxicam. Vet. Rec..

[38] Chang C.-L., Ma B., Pang X., Wu T.C., Hung C.-F. (2009). Treatment with cyclooxygenase-2 inhibitors enables repeated administration of vaccinia virus for control of ovarian cancer. Mol. Ther..

[39] Andrianaivoarimanana V., Kreppel K., Duplantier J.M., Carniel E., Rajerison M., Jambou R. (2013). Understanding the persistence of plague foci in Madagascar. PLoS Negl. Trop. Dis..

[40] Stenseth N.C., Atshabar B.B., Begon M., Belmain S.R., Bertherat E., Carniel E., Gage K.L., Leirs H., Rahalison L. (2008). Plague: Past, present, and future. PLoS Med..

[41] Andrianaivoarimanana V., Piola P., Wagner D.M., Rakotomanana F., Maheriniaina V., Andrianalimanana S., Chanteau S., Rahalison L., Ratsitorahina M., Rajerison M. (2019). Trends of human plague, madagascar, 1998–2016. Emerg. Infect. Dis..

[42] Randremanana R., Andrianaivoarimanana V., Nikolay B., Ramasindrazana B., Paireau J., Ten Bosch Q.A., Rakotondramanga J.M., Rahajandraibe S., Rahelinirina S., Rakotomanana F. (2019). Epidemiological characteristics of an urban plague epidemic in Madagascar, August-November, 2017: An outbreak report. Lancet Infect. Dis..

[43] Connor M.G., Pulsifer A.R., Chung D., Rouchka E.C., Ceresa B.K., Lawrenz M.B. (2018). Yersinia pestis targets the host endosome recycling pathway during the biogenesis of the Yersinia-containing vacuole to avoid killing by macrophages. MBio.

[44] Perry R.D., Fetherston J.D. (1997). *Yersinia pestis*—Etiologic agent of plague. Clin. Microbiol. Rev..

[45] Inglesby T.V., Dennis D.T., Henderson D.A., Batlett J.G., Ascher M.S., Eitzen E., Fine A.D., Friedlander A.M., Hauer J., Koerner J.F. (2000). Plague as a biological weapon: Medical and public health management. Work. Group Civ. Biodefense JAMA.

[46] Feodorova V.A., Motin V.L. (2018). Humoral and cellular immune responses to *Yersinia pestis* Pla antigen in humans immunized with live plague vaccine. PLoS Negl. Trop. Dis..

[47] Feodorova V.A., Motin V.L. (2012). Plague vaccines: Current developments and future perspectives. Emerg. Microbes Infect..

[48] Feodorova V.A., Sayapina L.V., Motin V.L. (2016). Assessment of live plague vaccine candidates. Methods Mol. Biol..

[49] Andersson J.A., Sha J., Erova T.E., Fitts E.C., Ponnusamy D., Kozlova E.V., Kirtley M.L., Chopra A.K. (2017). Identification of New Virulence Factors and Vaccine Candidates for *Yersinia pestis*. Front. Cell. Infect. Microbiol..

[50] Doll J.M., Zeitz P.S., Ettestad P., Bucholtz A.L., Davis T., Gage K. (1994). Cat-transmitted fatal pneumonic plague in a person who traveled from Colorado to Arizona. Am. J. Trop. Med. Hyg..

[51] Worsham P.L., Stein M.P., Welkos S.L. (1995). Construction of defined F1 negative mutants of virulent *Yersinia pestis*. Contrib. Microbiol. Immunol..

[52] Welkos S., Pitt M.L., Martinez M., Friedlander A., Vogel P., Tammariello R. (2002). Determination of the virulence of the pigmentation-deficient and pigmentation-/plasminogen activator-deficient strains of *Yersinia pestis* in non-human primate and mouse models of pneumonic plague. Vaccine.

[53] Welkos S.L., Friedlander A.M., Davis K.J. (1997). Studies on the role of plasminogen activator in systemic infection by virulent *Yersinia pestis* strain C092. Microb. Pathog..

[54] Jenkins A.L., Worsham P.L., Welkos S.L. (2009). A strategy to verify the absence of the pgm locus in *Yersinia pestis* strain candidates for select agent exemption. J. Microbiol. Methods.

[55] National Research Council (2011). Guide for the Care and Use of Laboratory Animals.

[56] Bachmanov A.A., Reed D.R., Beauchamp G.K., Tordoff M.G. (2002). Food intake, water intake, and drinking spout side preference of 28 mouse strains. Behav. Genet..

[57] Brunell M., Olsen C., Christy A., Maxwell B., Bentzel D. (2017). Evaluation of consumption of self-administered acetaminophen in drinking water and two gel delivery system in C57BL/6 mice. Internet J. Vet. Med..

[58] Kohn D.F., Wixson S.K., White W.J., Benson G.J. (2008). Anesthesia and Analgesia in Laboratory Animals.

[59] Mayer J., Mans C., Carpenter J.W., Marion C.J. (2018). Chapter 9-Rodents, in Exotic Animal Formulary.

[60] Amemiya K., Meyers J.L., Trevino S.R., Chanh T.C., Norris S.L., Waag D.M. (2006). Interleukin-12 induces a Th1-like response to *Burkholderia mallei* and limited protection in BALB/c mice. Vaccine.

[61] Bearss J.J., Hunter M., Dankmeyer J.L., Fritts K.A., Klimko C.P., Weaver C.H., Shoe J.L., Quirk A.V., Toothman R.G., Webster W.M. (2017). Characterization of pathogenesis of and immune response to *Burkholderia pseudomallei* K96243 using both inhalational and intraperitoneal infection models in BALB/c and C57BL/6 mice. PLoS ONE.

[62] Fetherston J.D., Perry R.D. (1994). The pigmentation locus of *Yersinia pestis* KIM6+ is flanked by an insertion sequence and includes the structural genes for pesticin sensitivity and HMWP2. Mol. Microbiol..

[63] Fetherston J.D., Schuetze P., Perry R.D. (1992). Loss of the pigmentation phenotype in *Yersinia pestis* is due to the spontaneous deletion of 102 kb of chromosomal DNA which is flanked by a repetitive element. Mol. Microbiol..

[64] Lucier T.S., Fetherston J.D., Brubaker R.R., Perry R.D. (1996). Iron uptake and iron-repressible polypeptides in *Yersinia pestis*. Infect. Immun..

[65] Ferber D.M., Brubaker R.R. (1981). Plasmids in *Yersinia pestis*. Infect. Immun..

[66] Sodeinde O.A., Subrahmanyam Y.V., Stark K., Quan T., Bao Y., Goguen J.D. (1992). A surface protease and the invasive character of plague. Science.

[67] Wilson S.G., Smith S.B., Chesler E.J., Melton K.A., Haas J.J., Mitton B., Strasburg K., Hubert L., Rodriguez-Zas S.L., Mogil J.S. (2003). The heritability of antinociception: common pharmacogenetic mediation of five neurochemically distinct analgesics. J. Pharmacol. Exp. Ther..

[68] Saharinen P., Tammela T., Karkkainen M.J., Alitalo K. (2004). Lymphatic vasculature: Development, molecular regulation and role in tumor metastasis and inflammation. Trends Immunol..

[69] Harrell M.I., Iritani B.M., Ruddell A. (2008). Lymph node mapping in the mouse. J. Immunol. Methods.

[70] Jiskoot W., Kijanka G., Randolph T.W., Carpenter J.F., Koulov A.V., Mahler H.-C., Joubert M.K., Jawa V., Narhi L.O. (2016). Mouse models for assessing protein immunogenicity: Lessons and challenges. J. Pharm. Sci..

[71] Morokata T., Ishikawa J., Yamada T. (2000). Antigen dose defines T helper 1 and T helper 2 responses in the lungs of C57BL/6 and BALB/c mice independently of splenic responses. Immunol. Lett..

[72] Dash B., Shapiro M.J., Chung J.Y., Romero-Arocha S., Shapiro V.S. (2018). Treg-specific deletion of NKAP results in severe, systemic autoimmunity due to peripheral loss of Tregs. J. Autoimmun..

[73] Hadaschik E.N., Wei X., Leiss H., Heckmann B., Niederreiter B., Steiner G., Ulrich W., Enk A.H., Smolen J.S., Stummvoll G.H. (2015). Regulatory T cell-deficient scurfy mice develop systemic autoimmune features resembling lupus-like disease. Arthritis Res. Ther..

[74] Lyon M.F., Peters J., Glenister P.H., Ball S., Wright E. (1990). The scurfy mouse mutant has previously unrecognized hematological abnormalities and resembles Wiskott-Aldrich syndrome. Proc. Natl. Acad. Sci. USA.

[75] Barthold S.W., Griffey S.M., Percy D.H. (2016). Pathology of Laboratory Rodents and Rabbits, 4th ed.

[76] Jirkof P. (2017). Side effects of pain and analgesia in animal experimentation. Lab. Anim..

[77] Sabhnani L., Rao D.N. (2000). Identification of immunodominant epitope of F1 antigen of *Yersinia pestis*. FEMS Immunol. Med. Microbiol..

[78] Williams R.C., Gewurz H., Quie P.G. (1972). Effects of fraction I from *Yersinia pestis* on phagocytosis in vitro. J. Infect. Dis..

[79] Friedlander A.M., Welkos S.L., Worsham P.L., Andrews G.P., Heath D.G., Anderson G.W., Pitt M.L., Estep J., Davis K. (1995). Relationship between virulence and immunity as revealed in recent studies of the F1 capsule of *Yersinia pestis*. Clin. Infect. Dis..

[80] Welkos S.L., Davis K.M., Pitt L.M., Worsham P.L., Friedlander A.M. (1995). Studies on the contribution of the F1 capsule-associated plasmid pFra to the virulence of *Yersinia pestis*. Contrib. Microbiol. Immunol..

[81] Martin R.M., Brady J.L., Lew A.M. (1998). The need for IgG2c specific antiserum when isotyping antibodies from C57BL/6 and NOD mice. J. Immunol. Methods.

[82] Morgado M.G., Cam P., Gris-Liebe C., Cazenave P.A., Jouvin-Marche E. (1989). Further evidence that BALB/c and C57BL/6 gamma 2a genes originate from two distinct isotypes. EMBO J..

[83] Engelhardt G. (1996). Pharmacology of meloxicam, a new non-steroidal anti-inflammatory drug with an improved safety profile through preferential inhibition of COX-2. Br. J. Rheumatol..

[84] Engelhardt G., Homma D., Schlegel K., Schnitzler C., Utzmann R. (1996). General pharmacology of meloxicam—Part II: Effects on blood pressure, blood flow, heart rate, ECG, respiratory minute volume and interactions with paracetamol, pirenzepine, chlorthalidone, phenprocoumon and tolbutamide. Gen. Pharmacol..

[85] Engelhardt G., Homma D., Schlegel K., Schnitzler C., Utzmann R. (1996). General pharmacology of meloxicam—Part I: Effects on CNS, gastric emptying, intestinal transport, water, electrolyte and creatinine excretion. Gen. Pharmacol..

[86] Bauer D.J., Christenson T.J., Clark K.R., Powell S.K., Swain R.A. (2003). Acetaminophen as a postsurgical analgesic in rats: A practical solution to neophobia. Contemp. Top. Lab. Anim. Sci..

[87] Guler M.L., Gorham J.D., Hsieh C.S., Mackey A.J., Steen R.G., Dietrich W.F., Murphy K.M. (1996). Genetic susceptibility to *Leishmania*: IL-12 responsiveness in TH1 cell development. Science.

[88] Reiner S.L., Locksley R.M. (1995). The regulation of immunity to *Leishmania major*. Annu. Rev. Immunol..

[89] Xu D., Trajkovic V., Hunter D., Leung B.P., Schulz K., Gracie J.A., McInnes I.B., Liew F.Y. (2000). IL-18 induces the differentiation of Th1 or Th2 cells depending upon cytokine milieu and genetic background. Eur. J. Immunol..

[90] Bohn E., Heesemann J., Ehlers S., Autenrieth I.B. (1994). Early gamma interferon mRNA expression is associated with resistance of mice against *Yersinia enterocolitica*. Infect. Immun..

[91] Ulett G.C., Ketheesan N., Hirst R.G. (2000). Cytokine gene expression in innately susceptible BALB/c mice and relatively resistant C57BL/6 mice during infection with virulent *Burkholderia pseudomallei*. Infect. Immun..

[92] Autenrieth I.B., Beer M., Bohn E., Kaufmann S.H., Heesemann J. (1994). Immune responses to *Yersinia enterocolitica* in susceptible BALB/c and resistant C57BL/6 mice: An essential role for gamma interferon. Infect. Immun..

[93] Kopf M., Le Gros G., Bachmann M., Lamers M.C., Bluethmann H., Kohler G. (1993). Disruption of the murine IL-4 gene blocks Th2 cytokine responses. Nature.

[94] Ishigame H., Nakajima A., Saijo S., Komiyama Y., Nambu A., Matsuki T., Nakae S., Horai R., Kakuta S., Iwakura Y. (2006). The role of TNFalpha and IL-17 in the development of excess IL-1 signaling-induced inflammatory diseases in IL-1 receptor antagonist-deficient mice. Ernst Schering Research Foundation Workshop.

[95] Iwakura Y., Ishigame H. (2006). The IL-23/IL-17 axis in inflammation. J. Clin. Investig..

[96] Chinen T., Kannan A.K., Levine A.G., Fan X., Klein U., Zheng Y., Gasteiger G., Feng Y., Fontenot J.D., Rudensky A.Y. (2016). An essential role for the IL-2 receptor in Treg cell function. Nat. Immunol..

[97] Malek T.R. (2003). The main function of IL-2 is to promote the development of T regulatory cells. J. Leukoc. Biol..

[98] El-Gowelli H.M., Helmy H.M., Ali R.W., El-Mas M.M. (2014). Celecoxib offsets the negative renal influences of cyclosporine via modulation of the TGF-beta1/IL-2/COX-2/endothelin ET(B) receptor cascade. Toxicol. Appl. Pharmacol..

[99] Hamada T., Tsuchihashi S., Avanesyan A., Duarte S., Moore C., Busuttil R.W., Coito A.J. (2008). Cyclooxygenase-2 deficiency enhances Th2 immune responses and impairs neutrophil recruitment in hepatic ischemia/reperfusion injury. J. Immunol..

[100] Laouini D., Elkhal A., Yalcindag A., Kawamoto S., Oettgen H., Geha R.S. (2005). COX-2 inhibition enhances the TH2 immune response to epicutaneous sensitization. J. Allergy Clin. Immunol..

[101] Nakajima S., Honda T., Sakata D., Egawa G., Tanizaki H., Otsuka A., Moniaga C.S., Watanabe T., Miyachi Y., Narumiya S. (2010). Prostaglandin I2-IP signaling promotes Th1 differentiation in a mouse model of contact hypersensitivity. J. Immunol..

[102] Lin J.S., Kummer L.W., Szaba F.M., Smiley S.T. (2011). IL-17 contributes to cell-mediated defense against pulmonary *Yersinia pestis* infection. J. Immunol..

[103] Basu S., Hodgson G., Katz M., Dunn A.R. (2002). Evaluation of role of G-CSF in the production, survival, and release of neutrophils from bone marrow into circulation. Blood.

[104] Bohle B., Kinaciyan T., Gerstmayr M., Radakovics A., Jahn-Schmid B., Ebner C. (2007). Sublingual immunotherapy induces IL-10-producing T regulatory cells, allergen-specific T-cell tolerance, and immune deviation. J. Allergy Clin. Immunol..

[105] Harizi H., Juzan M., Pitard V., Moreau J.F., Gualde N. (2002). Cyclooxygenase-2-issued prostaglandin e(2) enhances the production of endogenous IL-10, which down-regulates dendritic cell functions. J. Immunol..

[106] Hsu P., Santner-Nanan B., Hu M., Skarratt K., Lee C.H., Stormon M., Wong M., Fuller S.J., Nanan R. (2015). IL-10 Potentiates differentiation of human induced regulatory T Cells via STAT3 and Foxo1. J. Immunol..

[107] Quinton L.J., Mizgerd J.P., Hilliard K.L., Jones M.R., Kwon C.Y., Allen E. (2012). Leukemia inhibitory factor signaling is required for lung protection during pneumonia. J. Immunol..

[108] Nasef A., Mazurier C., Bouchet S., Ffrancois S., Chapel A., Thierry D., Gorin N.C., Fouillard L. (2008). Leukemia inhibitory factor: Role in human mesenchymal stem cells mediated immunosuppression. Cell. Immunol..

[109] Chen W.K., Ren L., Wei Y., Zhu D.X., Miao C.H., Xu J.M. (2015). General anesthesia combined with epidural anesthesia ameliorates the effect of fast-track surgery by mitigating immunosuppression and facilitating intestinal functional recovery in colon cancer patients. Int. J. Colorectal. Dis..

[110] Sofra M., Fei P.C., Fabrizi L., Marcelli M.E., Claroni C., Gallucci M., Ensoli F., Forastiere E. (2013). Immunomodulatory effects of total intravenous and balanced inhalation anesthesia in patients with bladder cancer undergoing elective radical cystectomy: Preliminary results. J. Exp. Clin. Cancer Res..

[111] Zhang S., Pan S.B., Lyu Q.H., Wu P., Qin G.M., Wang Q., He Z.L., He X.M., Wu M., Chen G. (2015). Postoperative regulatory T-Cells and natural killer cells in stage i nonsmall cell lung cancer underwent video-assisted thoracoscopic lobectomy or thoracotomy. Chin. Med. J..

[112] Ndure J., Noho-Konteh F., Adetifa J.U., Cox M., Barker F., Le M.T., Sanyang L.C., Drammeh A., Whittle H.C., Clarke E. (2017). Negative correlation between circulating CD+FOXP_3_+CD127- regulatory T cells and subsequent antibody responses to infant measles vaccine but not diphtheria-tetanus-pertussis vaccine implies regulatory role. Front. Immunol..

[113] Liu B., Zhao H., Poon M.C., Han Z., Gu D., Xu M., Jia H., Yang R., Han Z.C. (2007). Abnormality of CD4+CD25+ regulatory T cells in idiopathic thrombocytopenic purpura. Eur. J. Haematol..

[114] Miller E., Waight P., Farrington P., Andrews N., Stowe J., Taylor B. (2001). Idiopathic thrombocytopenic purpura and MMR vaccine. Arch. Dis. Child..

[115] Sakakura M., Wada H., Tawara I., Nobori T., Sugiyama T., Sagawa N., Shiku H. (2007). Reduced CD4+ CD25+ T cells in patients with idiopathic thrombocytopenic purpura. Thromb. Res..

[116] Attia P., Phan G.Q., Maker A.V., Robinson M.R., Quezado M.M., Yang J.C., Sherry R.M., Topalian S.L., Kammula U.S., Royal R.E. (2005). Autoimmunity correlates with tumor regression in patients with metastatic melanoma treated with anti-cytotoxic T-lymphocyte antigen-4. J. Clin. Oncol..

[117] Maker A.V., Attia P., Rosenberg S.A. (2005). Analysis of the cellular mechanism of antitumor responses and autoimmunity in patients treated with CTLA-4 blockade. J. Immunol..

[118] Maker A.V., Phan G.Q., Attia P., Yang J.C., Sherry R.M., Topalian S.L., Kammula U.S., Royal R.E., Haworth L.R., Levy C. (2005). Tumor regression and autoimmunity in patients treated with cytotoxic T lymphocyte-associated antigen 4 blockade and interleukin 2: A phase I/II study. Ann. Surg. Oncol..

[119] Daifalla N.S., Bayih A.G., Gedamu L. (2015). Differential Immune Response against Recombinant *Leishmania donovani* Peroxidoxin 1 and Peroxidoxin 2 Proteins in BALB/c Mice. J. Immunol. Res..

[120] Rostamian M., Sohrabi S., Kavosifard H., Niknam H.M. (2017). Lower levels of IgG1 in comparison with IgG2a are associated with protective immunity against *Leishmania tropica* infection in BALB/c mice. J. Microbiol. Immunol. Infect..

[121] Martin R.M., Silva A., Lew A.M. (1997). The Igh-1 sequence of the non-obese diabetic (NOD) mouse assigns it to the IgG2c isotype. Immunogenetics.

[122] Singh V.K., Mehrotra S., Agarwal S.S. (1999). The paradigm of Th1 and Th2 cytokines: Its relevance to autoimmunity and allergy. Immunol. Res..

[123] Snapper C.M., Peschel C., Paul W.E. (1988). IFN-gamma stimulates IgG2a secretion by murine B cells stimulated with bacterial lipopolysaccharide. J. Immunol..

[124] Heffernan J.M., Keeling M.J. (2009). Implications of vaccination and waning immunity. Proc. Biol. Sci..

[125] Ivins B.E., Fellows P.F., Pitt M.L.M., Estep J.E., Welkos S.L., Worsham P.L., Friedlander A.M. (1996). Efficacy of standard human anthrax vaccine against *Bacillus anthracis* aerosol spore challenge in rhesus monkeys. Salisb. Med Bull..

[126] Sarda V., Kaslow D.C., Williamson K.C. (2009). Approaches to malaria vaccine development using the retrospectroscope. Infect. Immun..

[127] Whittle H.C., Aaby P., Samb B., Jensen H., Bennett J., Simondon F. (1999). Effect of subclinical infection on maintaining immunity against measles in vaccinated children in West Africa. Lancet.

[128] Bowen W., Batra L., Pulsifer A.R., Yolcu E.S., Lawrenz M.B., Shirwan H. (2019). Robust Th1 cellular and humoral responses generated by the *Yersinia pestis* rF1-V subunit vaccine formulated to contain an agonist of the CD137 pathway do not translate into increased protection against pneumonic plague. Vaccine.

[129] Fink A.L., Engle K., Ursin R.L., Tang W.Y., Klein S. (2018). Biological sex affects vaccine efficacy and protection against influenza in mice. Proc. Natl. Acad. Sci. USA.

[130] Van Loo P.L., van Zutphen L.F., Baumans V. (2003). Male management: Coping with aggression problems in male laboratory mice. Lab. Anim..

[131] Kappel S., Hawkins P., Mendl M.T. (2017). To group or not to group? Good practice for housing male laboratory mice. Animals.

[132] Webster Marketon J.I., Glaser R. (2008). Stress hormones and immune function. Cell. Immunol..

[133] Averbuch M., Katzper M. (2000). A search for sex differences in response to analgesia. Arch. Intern. Med..

[134] Bartley E.J., Fillingim R.B. (2013). Sex differences in pain: A brief review of clinical and experimental findings. Br. J. Anaesth..

[135] Cho C., Michailidis V., Lecker I., Collymore C., Hanwell D., Loka M., Danesh M., Pham C., Urban P., Bonin L.J. (2019). Evaluating analgesic efficacy and administration route following craniotomy in mice using the grimace scale. Sci. Rep..

[136] Gioiosa L., Chen X., Watkins R., Umeda E.A., Arnold A.P. (2008). Sex chromosome complement affects nociception and analgesia in newborn mice. J. Pain.

[137] Greenspan J.D., Craft R.M., LeResche L., Arendt-Nielsen L., Berkley K.J., Fillingim R.B., Gold M.S., Holdcroft A., Lautenbacher S., Mayer E.A. (2007). Studying sex and gender differences in pain and analgesia: A consensus report. Pain.

[138] Rosen S.F., Ham B., Haichin M., Walters I.C., Tohyama S., Sotocinal S.G., Mogil J.S. (2019). Increased pain sensitivity and decreased opioid analgesia in T-cell-deficient mice and implications for sex differences. Pain.

[139] U.S. Department of Agriculture (2015). Animal Care Policy Manual: October 16, 2015 in Policy no. 11 (ACRG 11), Animal and Plant Health Inspection Service (APHIS).

[140] Denayer T., Stohr T., van Roy M. (2014). Animal models in translational medicine: Validation and prediction. New Horiz. Transl. Med..

[141] Gerdts V., Littel-van den Hurk S.V., Griebel P.J., Babiuk L.A. (2007). Use of animal models in the development of human vaccines. Future Microbiol..

[142] Sacerdote P. (2006). Opioids and the immune system. Palliat. Med..

[143] Cho J.Y. (2007). Immunomodulatory effect of nonsteroidal anti-inflammatory drugs (NSAIDs) at the clinically avalable doses. Arch. Pharm. Res..

[144] Kim H.J., Lee Y.H., Im S.A., Kim K., Lee C.K. (2010). Cyclooxygenase inhibitors, aspirin and ibuprofen, inhibit MHC-restricted antigen presentation in dendritic cells. Immune Netw..

