# Supplementary material for: The Use of Analgesics during Vaccination with a Live Attenuated Yersinia pestis Vaccine Alters the Resulting Immune Response in Mice
Source: Vaccines (Basel). 2019 Dec 3;7(4):205. doi: 10.3390/vaccines7040205 (PMC6963655; doi:10.3390/vaccines7040205)
Supplement: Supplementary file 1 [file vaccines-07-00205-s001.pdf]

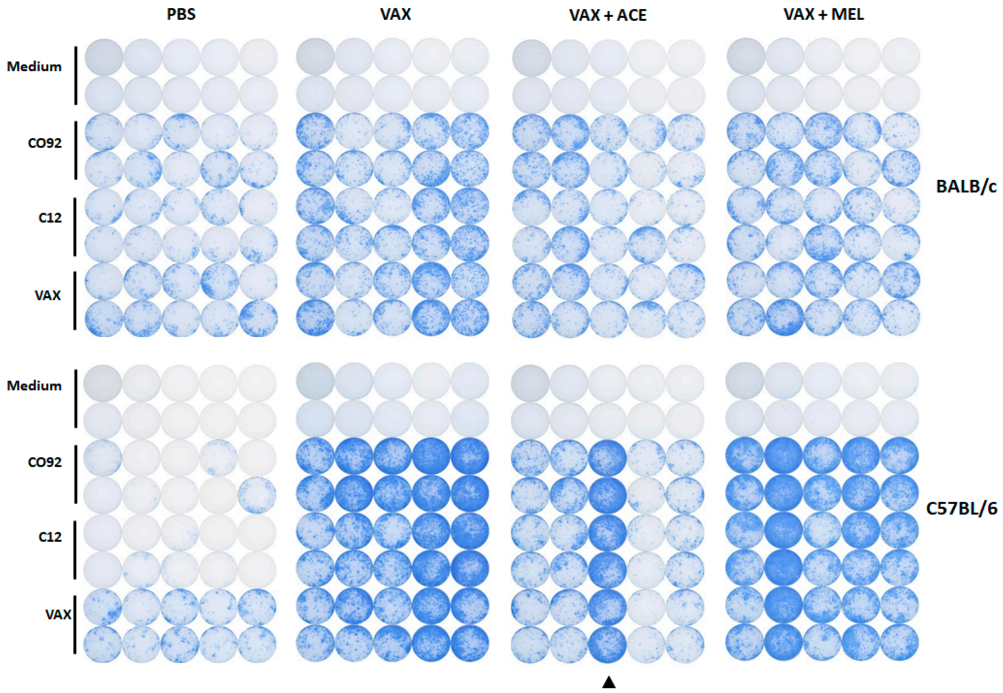

Figure S1. ELISpot assay well images of discreet IFN-γ secreting splenocytes on the membrane. Stimulation conditions are denoted on the left (Medium, *Y. pestis* CO92, *Y. pestis* C12, and *Y. pestis* VAX), mouse vaccine groups are denoted on top (PBS, VAX, VAX + ACE, and VAX + MEL), and mouse strains are denoted on the right (BALB/c and C57BL/6). Arrow denotes an outlier mouse from the C57BL/6 VAX + ACE group that was removed from further analysis.

Table S1. Change in weight from baseline in BALB/c mice through day 46 post vaccination +/- analgesia administration.

		BALB/C										
		VAX			VAX + ACE				VAX + MEL			
Treatment[a]	Day	n	Mean Change (SD) from baseline	% Weight Change	n	Mean Change (SD) from baseline	% Weight Change	vs. VAX (P-value)	n	Mean Change (SD) from baseline	% Weight Change	vs. VAX (P-value)
	-1	20	17.06 (0.22)[b]	n/a	20	17.56 (0.24)[b]	n/a	n/a	20	17.5 (0.18)[b]	n/a	n/a
Analgesia	0	20	0.25 (0.54)	1.47	20	0.18 (0.31)	1.03	ns	20	0.17 (0.28)	0.97	ns
VAX +/- Analgesia	1	20	0.35 (0.59)	2.05	20	0.37 (0.46)	2.11	ns	20	0.24 (0.33)	1.37	ns
Analgesia	2	20	-0.10 (0.80)	-0.59	20	-0.53 (0.55)	-3.02	< 0.05	20	-0.52 (0.45)	-2.97	< 0.05
	3	20	-1.25 (0.82)	-7.33	20	-1.77 (0.57)	-10.08	< 0.05	18	-1.52 (0.52)	-8.69	ns
	4	20	-2.22 (0.90)	-13.01	20	-2.52 (0.71)	-14.35	ns	18	-2.12 (0.89)	-12.11	ns
	5	20	-2.68 (1.14)	-15.71	20	-2.77 (1.06)	-15.77	ns	18	-2.33 (1.30)	-13.31	ns
	6	20	-2.87 (1.38)	-16.82	20	-2.64 (1.47)	-15.03	ns	18	-2.32 (1.51)	-13.26	ns
	7	20	-2.69 (1.69)	-15.77	19	-2.28 (1.68)	-12.98	ns	18	-1.81 (1.55)	-10.34	ns
	9	17	-1.09 (1.92)	-6.39	19	-1.17 (1.54)	-6.66	ns	18	-0.69 (1.52)	-3.94	ns
	11	17	0.02 (1.69)	0.12	19	0.27 (1.05)	1.54	ns	18	0.08 (1.53)	0.46	ns
	18	17	1.45 (1.83)	8.50	19	1.37 (0.85)	7.80	ns	18	1.13 (1.32)	6.46	ns
	25	17	2.64 (1.92)	15.47	19	2.60 (0.93)	14.81	ns	18	2.29 (0.97)	13.09	ns
Yp Challenge	29	-	-	-	-	-	-	-	-	-	-	-
	32	10	0.77 (1.23)	3.91	10	1.12 (0.71)	5.56	ns	10	0.57 (0.68)	2.88	ns
	42	10	2.14 (2.21)	10.86	10	1.11 (0.66)	5.51	ns	10	1.63 (0.64)	8.24	ns
	46	10	3.07 (0.51)	15.58	10	1.43 (0.87)	7.09	< 0.05	10	1.92 (0.51)	9.70	ns

a. Analgesia (ACE or MEL) was administered on day 0, 1, and 2. Mice were vaccinated on day 1 with Y. pestis pgm-/pPst- (VAX). Mice were challenged with Y. pestis (Yp) CO92 on day 28 post-vaccination (day 29).
b. The mean for the baseline weight (g/mouse) and standard deviation (SD) are shown on day -1, followed by the difference in mean weight (SD) from baseline on days 0 to 46.
Abbreviation: n/a, not applicable; ns, not significant.

Table S2. Change in weight from baseline in C57BL/6 mice through day 46 post vaccination +/- analgesia administration.

Treatment[a]	Day	C57BL/6 VAX n	VAX Mean Change (SD) from baseline	VAX % Weight Change	VAX + ACE n	VAX + ACE Mean Change (SD) from baseline	VAX + ACE % Weight Change	VAX + ACE vs. VAX (P-value)	VAX + MEL n	VAX + MEL Mean Change (SD) from baseline	VAX + MEL % Weight Change	VAX + MEL vs. VAX (P-value)
	-1	20	17.29 (0.19)[b]	n/a	20	17.96 (0.48)[b]	n/a	n/a	20	17.84 (0.29)[b]	n/a	n/a
Analgesia	0	20	0.38 (0.36)	2.20	20	-0.06 (0.34)	-0.34	< 0.05	20	-0.03 (0.3)	-0.17	< 0.05
VAX +/- Analgesia	1	20	0.59 (0.34)	3.41	20	0.12 (0.29)	0.68	< 0.05	20	0.01 (0.38)	0.06	< 0.05
Analgesia	2	20	-1.72 (0.46)	-9.95	20	-1.53 (0.61)	-8.65	ns	20	-1.78 (0.56)	-9.98	ns
	3	20	-2.04 (0.41)	-11.80	20	-1.89 (0.57)	-10.68	ns	18	-1.91 (0.66)	-10.71	ns
	4	20	-2.18 (0.44)	-12.61	20	-2.18 (0.62)	-12.32	ns	17	-2.02 (0.68)	-11.32	ns
	5	20	-2.07 (0.83)	-11.97	20	-2.03 (0.81)	-11.48	ns	17	-1.72 (0.88)	-9.64	ns
	6	20	-2.09 (1.27)	-12.09	20	-2.00 (1.33)	-11.31	ns	17	-1.36 (0.93)	-7.62	< 0.05
	7	20	-1.85 (1.76)	-10.70	20	-1.39 (1.45)	-7.86	ns	17	-0.68 (0.74)	-3.81	< 0.05
	9	20	-0.91 (1.91)	-5.26	20	0.04 (1.60)	0.23	< 0.05	17	0.07 (0.58)	0.39	< 0.05
	11	20	-0.32 (1.43)	-1.85	20	0.32 (1.42)	1.81	ns	17	0.35 (0.52)	1.96	ns
	18	19	1.38 (1.05)	7.98	20	1.42 (1.21)	8.03	ns	17	1.15 (1.01)	6.45	ns
	25	19	2.67 (1.31)	15.44	20	2.33 (1.16)	13.17	ns	17	2.08 (0.85)	11.66	ns
Yp Challenge	29	-	-	-	-	-	-	-	-	-	-	-
	32	10	0.76 (0.49)	4.07	10	1.45 (0.66)	7.24	< 0.05	10	1.01 (0.44)	5.07	ns
	42	10	1.15 (0.66)	6.16	10	1.62 (1.22)	8.09	ns	10	1.51 (1.04)	7.58	ns
	46	10	1.68 (0.69)	9.00	10	2.05 (0.93)	10.24	ns	10	2.08 (0.85)	10.44	ns

a. Analgesia (ACE or MEL) was administered on day 0, 1, and 2. Mice were vaccinated on day 1 with Y. pestis pgm-/pPst- (VAX). Mice were challenged with Y. pestis (Yp) CO92 on day 28 post-vaccination (day 29).
b. The mean for the baseline weight (g/mouse) and standard deviation (SD) are shown on day -1, followed by the difference in mean weight (SD) from baseline on days 0 to 46.
Abbreviation: n/a, not applicable; ns, not significant.

Table S3. Total food and water consumption rates (Combined data for BALB/c and C57BL/6 mice).

Treatment[b]	Day[c]	Pans[d]	VAX[a] Water Mean (SD)[e]	VAX[a] Food Mean (SD)[e]	VAX + ACE[a] Water Mean (SD)[e]	vs. VAX (P-value)	VAX + ACE[a] Food Mean (SD)[e]	vs. VAX (P-value)	VAX + MEL[a] Water Mean (SD)[e]	vs. VAX (P-value)	VAX + MEL[a] Food Mea (SD)[e]
	-1	4	3.30 (0.27)	1.95 (1.31)	3.71 (0.97)	ns	2.49 (0.21)	ns	3.59 (0.27)	ns	2.78 (0.36
Analgesia	0	4	3.39 (0.59)	3.20 (1.15)	3.52 (0.56)	ns	2.51 (0.09)	< 0.05	3.83 (1.23)	ns	2.52 (0.15
VAX +/- Analgesia	1	4	3.96 (0.61)	2.60 (0.14)	4.79 (0.46)	ns	2.58 (0.12)	ns	4.01 (0.48)	ns	2.71 (0.19
Analgesia	2	4	1.91 (0.10)	1.46 (0.63)	3.26 (1.70)	< 0.05	1.62 (0.35)	ns	2.49 (0.50)	ns	1.56 (0.42
	3	4	3.34 (0.43)	0.62 (0.13)	3.05 (0.63)	ns	0.58 (0.18)	ns	3.58 (0.77)	ns	0.97 (0.33
	4	4	2.11 (0.46)	0.61 (0.31)	1.94 (0.33)	ns	0.68 (0.29)	ns	4.99 (1.14)	< 0.05	0.89 (0.30
	5	4	2.12 (0.45)	0.98 (0.25)	2.13 (0.41)	ns	1.05 (0.30)	ns	2.84 (0.46)	ns	1.22 (0.25
	6	4	1.58 (0.27)	1.17 (0.20)	1.62 (0.38)	ns	1.46 (0.40)	ns	2.16 (0.29)	ns	1.75 (0.47

a. In order to achieve greater statistical power the water and food consumption data were combined for both BALB/c and C57BL/6 mice receiving the same vaccine and treatment regimens.
b. Analgesia (ACE or MEL) was administered on day 0, 1, and 2. Mice were vaccinated on day 1 with Y. pestis pgm-/pPst- (VAX).
c. Weights of food and water consumed were recoded daily starting on day -1 and ending on day 6.
d. Used to calculate the per capita food or water consumption in each pan.
e. Food and water consumption mean and standard deviation (SD) based on the values from 4 separate pans per vaccine group.
Abbreviation: ns, not significant.

Table S4. Food consumption rates (BALB/c and C57BL/6 mice).

Treatment[a]	Day[b]	Pans[c]	VAX C57BL/6[d]	VAX BALB/c[d]	VAX + ACE C57BL/6[d]	VAX + ACE BALB/c[d]	VAX + MEL C57BL/6[d]	VAX + MEL BALB/c[d]
	-1	2	2.73 (0.01)	1.18 (1.66)*	2.66 (0.12)	2.32 (0.01)	3.03 (0.37)	2.53 (0.04)
Analgesia	0	2	2.81 (0.04)	3.59 (1.83)	2.53 (0.16)	2.49 (0.01)	2.52 (0.26)	2.52 (0.04)
VAX +/- Analgesia	1	2	2.70 (0.04)	2.49 (0.12)	2.53 (0.05)	2.63 (0.18)	2.84 (0.13)	2.58 (0.17)
Analgesia	2	2	0.93 (0.17)	2.00 (0.00)	1.46 (0.50)	1.78 (0.11)	1.23 (0.21)	1.90 (0.16)*
	3	2	0.70 (0.11)	0.55 (0.12)	0.73 (0.11)	0.43 (0.04)	1.18 (0.32)	0.77 (0.24)
	4	2	0.88 (0.03)	0.34 (0.06)	0.91 (0.06)	0.45 (0.18)	1.09 (0.17)	0.68 (0.27)
	5	2	1.17 (0.18)	0.80 (0.13)	1.05 (0.04)	1.04 (0.52)	1.38 (0.19)	1.06 (0.23)
	6	2	1.28 (0.22)	1.05 (0.09)	1.41 (0.45)	1.51 (0.53)	2.02 (0.52)	1.48 (0.31)

a. Analgesia (ACE or MEL) was administered on day 0, 1, and 2. Mice were vaccinated on day 1 with *Y. pestis* pgm-/pPst- (VAX).
b. Weights of food consumed were recoded daily starting on day -1 and ending on day 6.
c. Used to calculate the per capita food or water consumption in each pan.
d. Food consumption mean and standard deviation (SD) based on the values from 2 separate pans per vaccine group.
* Significant difference between mouse strains.

Table S5. Water consumption rates (BALB/c and C57BL/6 mice).

Treatment[a]	Day[b]	Pans[c]	VAX C57BL/6[d]	VAX BALB/c[d]	VAX + ACE C57BL/6[d]	VAX + ACE BALB/c[d]	VAX + MEL C57BL/6[d]	VAX + MEL BALB/c[d]
	-1	2	3.49 (0.26)	3.11 (0.04)	3.40 (0.04)	4.01 (1.56)	3.66 (0.04)	3.52 (0.44)
Analgesia	0	2	3.59 (0.17)	3.18 (0.92)	3.39 (0.11)	3.66 (0.93)	4.67 (1.33)	2.99 (0.02)*
VAX +/- Analgesia	1	2	4.43 (0.38)	3.49 (0.31)*	4.96 (0.72)	4.63 (0.13)	4.08 (0.04)	3.94 (0.81)
Analgesia	2	2	1.94 (0.13)	1.88 (0.10)	4.58 (1.22)	1.93 (0.40)*	2.06 (0.05)	2.92 (0.09)
	3	2	3.15 (0.44)	3.53 (0.47)	3.32 (0.54)	2.79 (0.79)	3.70 (0.46)	3.46 (1.23)
	4	2	2.48 (0.19)	1.73 (0.13)*	2.16 (0.16)	1.73 (0.36)	5.28 (1.32)	4.71 (1.34)
	5	2	2.46 (0.23)	1.79 (0.34)	2.07 (0.20)	2.19 (0.68)	2.85 (0.69)	2.83 (0.39)
	6	2	1.73 (0.21)	1.43 (0.30)	1.67 (0.48)	1.57 (0.44)	2.23 (0.28)	2.08 (0.40)

a. Analgesia (ACE or MEL) was administered on day 0, 1, and 2. Mice were vaccinated on day 1 with Y. pestis pgm-/pPst- (VAX).
b. Volumes of water consumed were recoded daily starting on day -1 and ending on day 6.
c. Used to calculate the per capita food or water consumption in each pan.
d. Water consumption mean and standard deviation (SD) based on the values from 2 separate pans per vaccine group.
* Significant difference between mouse strains.

Table S6. ELISpot IFN-γ responses in VAX vaccinated BALB/c and C57BL/6 mice treated concurrently with analgesia.

		C12				CO92				VAX			
		n	Median[a]	Geo Mean (GSE)[a]	vs. VAX (P-Value)[b]	n	Median[a]	Geo Mean (GSE)[a]	vs. VAX (P-Value)[b]	n	Median[a]	Geo Mean (GSE)[a]	vs. VAX (P-Value)[b]
BALB/c	PBS	5	1,674	1,543 (1.11)	0.0001	5	1,775	1,626 (1.12)	0.0034	5	2,804	2,863 (1.13)	0.0120
	VAX	5	8,122	6,483 (1.25)		5	7,436	5,519 (1.30)		5	8,108	6,746 (1.33)	
	VAX + ACE	5	2,081	2,540 (1.24)	0.0061	5	3,438	3,807 (1.32)	0.3268	5	3,690	3,248 (1.23)	0.0287
	VAX + MEL	5	3,706	3,295 (1.12)	0.0385	5	4,176	4,455 (1.22)	0.5687	5	4,406	4,739 (1.08)	0.2683
C57BL/6	PBS	5	1	5 (2.29)	<.0001	5	13	10 (1.73)	<.0001	5	3,269	2,741 (1.19)	<.0001
	VAX	5	12,591	13,689 (1.22)		5	21,362	20,020 (1.09)		5	12,190	13,704 (1.17)	
	VAX + ACE[c]	4	4,104	2,922 (1.62)	0.0410	4	4,116	3,993 (1.41)	0.0022	4	3,188	2,280 (1.51)	<.0001
	VAX + MEL	5	14,471	12,323 (1.23)	0.9262	5	18,985	18,926 (1.08)	0.8965	5	10,436	12,115 (1.16)	0.6997

a. Median and Geo Mean are reported as spot forming cells (SFC)/10^6 cells.

b. P-values reflect the result of post-hoc comparisons under a repeated measures ANOVA model.

c. Table excludes animal 3 of VAX + ACE C57BL/6 group.

Table S7. Comparison of analgesia treatment on the cytokine response in VAX vaccinated BALB/c mice.

			PBS			VAX					VAX + ACE					VAX + MEL				
Cytokine	Stimulation	ANOVA F-test (P-Value)[a]	n	Median	Geo Mean[b] (GSE)	n	Median	Geo Mean[b] (GSE)	GMFR to PBS[c]	vs. PBS (P-Value)[d]	n	Median	Geo Mean[b] (GSE)	GMFR to PBS[c]	vs. PBS (P-Value)[d]	n	Median	Geo Mean[b] (GSE)	GMFR to PBS[c]	vs. PBS (P-Value)[d]
G-CSF/CSF-3	CO92	0.0245	5	53.8	43.11 (1.31)	5	135.5	143.88 (1.19)	3.34	0.0072	5	139.5	147.93 (1.16)	3.43	0.0064	5	173.3	147.21 (1.23)	3.41	0.0075
	VAX	0.0052	5	56.9	62.14 (1.12)	5	122.4	125.96 (1.17)	2.03	0.0085	5	142.8	136.34 (1.14)	2.19	0.0019	5	149.9	122.87 (1.18)	1.98	0.0115
IL-10	CO92	0.0823	5	26.2	21.49 (1.39)	5	54.3	61.08 (1.19)	2.84	0.0297	5	60.6	61.42 (1.08)	2.86	0.0305	5	67.2	68.89 (1.13)	3.21	0.0198
	VAX	0.001	5	25.3	25.67 (1.03)	5	43.8	48.69 (1.18)	1.90	0.0154	5	52.8	57.01 (1.11)	2.22	0.0011	5	47.7	44.61 (1.10)	1.74	0.0033
IL-13	CO92	0.004	5	22.9	18.60 (1.30)	5	101.4	100.65 (1.29)	5.41	0.0018	5	125.1	110.02 (1.17)	5.92	0.0009	5	73.0	87.97 (1.21)	4.73	0.0019
	VAX	0.0002	5	30.2	30.03 (1.09)	5	117.2	110.16 (1.24)	3.67	0.0020	5	141.9	120.15 (1.20)	4	0.0006	5	77.4	85.39 (1.24)	2.84	0.0055
IL-17A	CO92	0.0081	5	18.0	27.84 (1.93)	5	1388.1	1376.74 (1.45)	49.45	0.0018	5	1161.1	979.77 (1.25)	35.19	0.0039	5	1242.1	1332.83 (1.35)	47.87	0.0021
	VAX	0.0011	5	89.2	79.03 (1.44)	5	1525.1	1366.28 (1.48)	17.29	0.0007	5	1635.1	1273.09 (1.29)	16.11	0.0004	5	1918.1	1577.38 (1.39)	19.96	0.0003
IL-2	CO92	0.0007	5	23.8	28.70 (1.22)	5	222.1	214.87 (1.38)	7.49	0.0013	5	156.1	133.69 (1.25)	4.66	0.0008	5	104.4	123.38 (1.24)	4.3	0.0011
	VAX	0.0003	5	24.8	29.10 (1.16)	5	206.4	196.50 (1.46)	6.75	0.0046	5	145.1	133.44 (1.23)	4.59	0.0004	5	95.0	113.86 (1.23)	3.91	0.0009
IL-22	CO92	0.1191	5	1168.4	820.86 (1.67)	5	3178.3	3399.04 (1.15)	4.14	0.0479	5	3975.0	3892.36 (1.06)	4.74	0.0379	5	4007.4	3719.65 (1.06)	4.53	0.0413
	VAX	0.0012	5	1863.5	1916.03 (1.11)	5	3056.9	3551.10 (1.13)	1.85	0.0052	5	4440.0	4550.24 (1.11)	2.37	0.0004	5	5001.2	4570.29 (1.10)	2.39	0.0003
IL-23	CO92	0.0458	5	22.8	19.73 (1.13)	5	29.6	31.67 (1.10)	1.61	0.0163	5	35.9	32.59 (1.11)	1.65	0.0148	5	34.3	32.75 (1.11)	1.66	0.0141
	VAX	0.0094	5	22.8	23.28 (1.10)	5	34.4	30.35 (1.19)	1.30	0.2372	5	45.0	38.51 (1.14)	1.65	0.0179	5	39.0	38.53 (1.07)	1.65	0.0037
IL-27	CO92	0.0013	5	2.8	3.12 (1.15)	5	6.6	7.46 (1.17)	2.39	0.0038	5	9.0	9.40 (1.06)	3.01	0.0007	5	9.1	9.15 (1.06)	2.93	0.0008
	VAX	0.0004	5	4.1	4.27 (1.09)	5	6.6	7.32 (1.17)	1.71	0.0207	5	10.8	10.43 (1.14)	2.44	0.0009	5	10.1	9.30 (1.09)	2.18	0.0002
IL-3	CO92	0.0026	5	5.9	6.19 (1.28)	5	25.7	34.12 (1.50)	5.51	0.0096	5	35.8	29.48 (1.24)	4.76	0.0016	5	30.1	28.87 (1.18)	4.66	0.0014
	VAX	0.0002	5	7.3	7.23 (1.10)	5	23.1	33.24 (1.50)	4.60	0.0184	5	35.6	29.42 (1.20)	4.07	0.0005	5	29.6	28.39 (1.12)	3.93	<.0001
IL-4	CO92	<.0001	5	3.2	3.10 (1.14)	5	22.8	32.72 (1.70)	10.56	0.0099	5	31.1	30.27 (1.06)	9.77	<.0001	5	30.8	29.63 (1.19)	9.56	<.0001
	VAX	<.0001	5	3.8	3.59 (1.10)	5	29.0	42.12 (1.66)	11.73	0.0073	5	31.8	34.09 (1.08)	9.49	<.0001	5	32.6	31.40 (1.14)	8.74	<.0001
IL-5	CO92	0.0038	5	12.9	11.89 (1.18)	5	33.5	33.06 (1.12)	2.78	0.0015	5	41.4	39.83 (1.08)	3.35	0.0009	5	33.2	34.81 (1.08)	2.93	0.0015
	VAX	<.0001	5	18.2	18.23 (1.06)	5	36.9	35.43 (1.14)	1.94	0.0051	5	46.9	42.75 (1.07)	2.34	<.0001	5	35.7	36.34 (1.07)	1.99	<.0001
IL-9	CO92	0.0174	5	29.8	28.31 (1.44)	5	169.2	188.48 (1.16)	6.66	0.0041	5	184.4	180.29 (1.07)	6.37	0.0062	5	180.8	181.04 (1.10)	6.4	0.0057
	VAX	0.0035	5	54.9	55.32 (1.21)	5	177.8	187.92 (1.17)	3.40	0.0012	5	216.4	203.85 (1.10)	3.68	0.0009	5	224.4	204.78 (1.10)	3.7	0.0008
LIF	CO92	0.0006	5	7.0	6.70 (1.18)	5	24.5	24.66 (1.23)	3.68	0.0014	5	29.7	26.63 (1.14)	3.98	0.0002	5	22.6	24.36 (1.09)	3.64	0.0004
	VAX	0.0003	5	9.2	8.36 (1.14)	5	23.7	22.13 (1.25)	2.65	0.0088	5	25.7	25.77 (1.09)	3.08	0.0002	5	26.1	25.23 (1.10)	3.02	0.0002
M-CSF	CO92	<.0001	5	0.8	0.69 (1.06)	5	1.1	1.03 (1.14)	1.49	0.0389	5	1.5	1.45 (1.04)	2.09	<.0001	5	1.6	1.37 (1.12)	1.97	0.0015
	VAX	0.0077	5	0.8	0.71 (1.12)	5	0.9	0.92 (1.11)	1.30	0.1375	5	1.4	1.42 (1.09)	2.01	0.0017	5	1.3	1.19 (1.11)	1.69	0.0105

a. One-way ANOVA test of equality of geometric means across treatment groups. P-Values in red failed to show statistical significance at the 0.05 level.

b. Cytokine levels are given in **pg/mL**, and the geometric mean (Geo Mean) and geometric standard error (GSE) are shown.

c. Geometric mean fold rise (GMFR) relative to PBS vaccinated group.

d. P-value indicates the result of Welch's t-test on log transformed values.

Table S8. Comparison of analgesia treatment on the cytokine response in VAX vaccinated C57BL/6 mice.

			PBS			VAX					VAX + ACE					VAX + MEL				
Cytokine	Stimulation	ANOVA F-test (P-Value)[a]	n	Median	Geo Mean (GSE)[b]	n	Median	Geo Mean (GSE)[b]	GMFR to PBS[c]	vs. PBS (P-Value)[d]	n	Median	Geo Mean (GSE)[b]	GMFR to PBS[c]	vs. PBS (P-Value)[d]	n	Median	Geo Mean (GSE)[b]	GMFR to PBS[c]	vs. PBS (P-Value)[d]
G-CSF/CSF-3	CO92	0.0300	5	82.3	86.70 (1.12)	5	153.3	164.00 (1.12)	1.89	0.0041	4	129.8	130.38 (1.12)	1.50	0.0384	5	133.9	128.86 (1.11)	1.49	0.0338
	VAX	0.0687	5	81.6	85.55 (1.16)	5	156.8	160.00 (1.12)	1.87	0.0107	4	118.9	124.86 (1.12)	1.46	0.0811	5	137.4	130.46 (1.12)	1.53	0.0573
IL-10	CO92	0.0034	5	46.9	44.20 (1.14)	5	114.6	121.08 (1.10)	2.74	0.0004	4	94.4	92.27 (1.10)	2.09	0.0029	5	107.0	104.41 (1.14)	2.36	0.0018
	VAX	0.0005	5	31.4	33.39 (1.07)	5	73.0	79.32 (1.13)	2.38	0.0007	4	64.6	61.78 (1.11)	1.85	0.0037	5	64.3	69.00 (1.15)	2.07	0.0043
IL-13	CO92	<.0001	5	15.2	14.79 (1.14)	5	107.1	112.25 (1.30)	7.59	0.0005	4	91.9	89.43 (1.15)	6.04	<.0001	5	81.5	90.50 (1.26)	6.12	0.0004
	VAX	0.0001	5	21.2	19.81 (1.15)	5	111.0	109.59 (1.27)	5.53	0.0007	4	105.2	98.66 (1.23)	4.98	0.0008	5	94.5	87.40 (1.17)	4.41	0.0001
IL-17A	CO92	<.0001	5	4.1	4.08 (1.37)	5	613.8	523.95 (1.59)	128.55	<.0001	4	429.2	416.41 (1.23)	102.16	<.0001	5	521.9	575.51 (1.39)	141.20	<.0001
	VAX	<.0001	5	8.5	11.77 (1.42)	5	565.1	521.28 (1.63)	44.30	0.0003	4	507.1	478.06 (1.24)	40.63	<.0001	5	416.8	513.50 (1.37)	43.64	<.0001
IL-2	CO92	<.0001	5	18.3	17.13 (1.07)	5	74.7	85.26 (1.26)	4.98	0.0015	4	65.4	65.52 (1.13)	3.83	0.0003	5	91.6	83.34 (1.16)	4.87	<.0001
	VAX	0.0004	5	13.3	13.54 (1.04)	5	63.8	68.76 (1.28)	5.08	0.0023	4	55.1	56.99 (1.17)	4.21	0.0020	5	65.0	62.08 (1.13)	4.58	<.0001
IL-22	CO92	0.0009	5	732.0	728.28 (1.22)	5	4335.3	3979.88 (1.22)	5.46	0.0003	4	3354.6	3261.68 (1.27)	4.48	0.0026	5	4271.1	3932.22 (1.16)	5.40	0.0002
	VAX	0.0054	5	1301.9	1584.03 (1.20)	5	4866.0	4505.99 (1.17)	2.84	0.0024	4	4558.9	4732.00 (1.20)	2.99	0.0037	5	4886.5	5152.42 (1.13)	3.25	0.0009
IL-23	CO92	0.0236	5	12.2	12.33 (1.21)	5	31.2	29.37 (1.13)	2.38	0.0068	4	35.8	31.65 (1.11)	2.57	0.0049	5	32.8	27.16 (1.19)	2.20	0.0158
	VAX	0.0252	5	16.0	17.59 (1.17)	5	29.5	31.31 (1.16)	1.78	0.0279	4	40.5	37.83 (1.09)	2.15	0.0049	5	29.5	30.40 (1.11)	1.73	0.0226
IL-27	CO92	0.0276	5	6.3	5.23 (1.23)	5	13.4	12.87 (1.11)	2.46	0.0080	4	8.8	9.10 (1.13)	1.74	0.0578	5	12.0	11.89 (1.07)	2.27	0.0131
	VAX	0.0368	5	8.0	7.06 (1.17)	5	13.4	12.69 (1.07)	1.80	0.0167	4	11.6	11.19 (1.16)	1.58	0.0723	5	13.2	13.80 (1.04)	1.95	0.0112
IL-3	CO92	0.0004	5	5.3	5.41 (1.09)	5	22.8	22.01 (1.34)	4.07	0.0067	4	18.2	18.44 (1.34)	3.41	0.0208	5	18.7	19.35 (1.17)	3.58	0.0003
	VAX	0.0007	5	4.4	4.80 (1.10)	5	23.1	19.31 (1.39)	4.02	0.0109	4	17.2	17.57 (1.36)	3.66	0.0201	5	15.3	16.41 (1.15)	3.42	0.0001
IL-4	CO92	<.0001	5	4.5	4.73 (1.16)	5	36.6	37.72 (1.27)	7.97	0.0002	4	24.1	27.89 (1.20)	5.89	0.0003	5	34.0	29.81 (1.22)	6.30	0.0001
	VAX	<.0001	5	5.2	5.47 (1.16)	5	43.3	42.13 (1.24)	7.70	<.0001	4	30.3	32.95 (1.24)	6.02	0.0006	5	35.0	30.51 (1.19)	5.58	<.0001
IL-5	CO92	0.0007	5	19.3	18.44 (1.08)	5	42.9	43.62 (1.10)	2.37	0.0001	4	32.4	31.05 (1.13)	1.68	0.0161	5	35.9	34.50 (1.12)	1.87	0.0028
	VAX	0.0041	5	17.0	19.10 (1.10)	5	36.1	37.86 (1.10)	1.98	0.0012	4	35.9	36.05 (1.07)	1.89	0.0013	5	36.3	37.06 (1.07)	1.94	0.0008
IL-9	CO92	<.0001	5	18.1	15.43 (1.13)	5	164.4	171.54 (1.19)	11.12	<.0001	4	155.6	152.24 (1.21)	9.87	0.0001	5	163.1	175.34 (1.14)	11.36	<.0001
	VAX	0.0046	5	26.0	25.73 (1.36)	5	178.5	177.44 (1.18)	6.89	0.0014	4	173.5	179.50 (1.18)	6.97	0.0015	5	182.9	191.48 (1.12)	7.44	0.0016
LIF	CO92	0.0010	5	8.4	6.83 (1.19)	5	29.3	32.37 (1.16)	4.74	0.0002	4	22.9	20.83 (1.15)	3.05	0.0016	5	28.8	30.25 (1.12)	4.43	0.0002
	VAX	<.0001	5	7.8	8.09 (1.10)	5	32.7	29.50 (1.17)	3.65	0.0003	4	19.1	19.92 (1.16)	2.46	0.0034	5	32.2	31.47 (1.11)	3.89	<.0001
M-CSF	CO92	0.0014	5	0.9	0.85 (1.23)	5	1.9	1.92 (1.06)	2.26	0.0141	4	1.1	1.09 (1.04)	1.28	0.2926	5	1.5	1.46 (1.07)	1.72	0.0547
	VAX	0.0153	5	0.6	0.72 (1.26)	5	1.7	1.78 (1.09)	2.45	0.0143	4	1.0	1.05 (1.09)	1.45	0.1947	5	1.4	1.30 (1.09)	1.79	0.0636

a. One-way ANOVA test of equality of geometric means across treatment groups. P-Values in red failed to show statistical significance at the 0.05 level.

b. Cytokine levels are given in **pg/mL**, and the geometric mean (Geo Mean) and geometric standard error (GSE) are shown.

c. Geometric mean fold rise (GMFR) relative to PBS vaccinated group.

d. P-value indicates the result of Welch's t-test on log transformed values.

Table S9. Total IgG titers determined by ELISA in sera collected from mice 28 days post vaccination +/- analgesia.

			CO92[a]			VAX[a]		
		**n**	**Median**	**Geo Mean (GSE)**	**vs. VAX (P-Value)[b]**	**Median**	**Geo Mean (GSE)**	**vs. VAX (P-Value)[b]**
**BALB/c**	**PBS**	9	50	50.0 (1.00)	<0.0001	50	50.0 (1.00)	<0.0001
	**VAX**	7	3,200	2,625.1 (1.80)		6,400	4,914.8 (1.75)	
	**VAX + ACE**	9	2,540	2,475.5 (1.24)	0.9260	4,032	4,136.6 (1.16)	0.7685
	**VAX + MEL**	8	2,851	2,198.3 (1.49)	0.8050	4,032	3,591.9 (1.42)	0.6391
**C57BL/6**	**PBS**	9	50	50.0 (1.00)	<0.0001	50	77.4 (1.46)	<0.0001
	**VAX**	9	12,800	5,486.4 (1.77)		16,127	13,132.9 (1.41)	
	**VAX + ACE**	10	4,525	3,509.8 (1.53)	0.5360	12,800	10,159.4 (1.24)	0.5323
	**VAX + MEL**	7	5,080	3,900.8 (1.56)	0.6418	16,127	14,606.6 (1.44)	0.8337

a. Irradiated CO92 or irradiated VAX (Y. pestis CO92 pgm-/pPst-) cells were used as capture antigen at a concentration of 10 µg/ml.
b. P-values reflect the result of post-hoc comparisons under a repeated measures ANOVA model.

Table S10. IgG1 and IgG2a/c titers determined by ELISA in sera collected from mice 28 days post vaccination +/- analgesia.

			CO92[a]					VAX[a]				
			IgG1		IgG2a/c[b]			IgG1		IgG2a/c[b]		
		**n**	**Geo Mean (GSE)**	**vs. VAX (P-Value)[c]**	**Geo Mean (GSE)**	**vs. VAX (P-Value)[c]**	**Ratio IgG2a/c / IgG1**	**Geo Mean (GSE)**	**vs. VAX (P-Value)[c]**	**Geo Mean (GSE)**	**vs. VAX (P-Value)[c]**	**Ratio IgG2a/c / IgG1**
**BALB/c**	**PBS**	9	50.0 (1.00)	≤0.0001	50.0 (1.00)	0.0125	1.000	50.0 (1.00)	≤0.0001	50.0 (1.00)	0.0026	1.000
	**VAX**	7	7,740.3 (1.66)		307.2 (1.97)		0.040	11,982.3 (1.66)		614.3 (2.13)		0.051
	**VAX + ACE**	9	7,407.0 (1.31)	0.9396	50.0 (1.00)	0.0125	0.007	20,847.1 (1.19)	0.308	90.2 (1.36)	0.0258	0.004
	**VAX + MEL**	8	8,234.4 (1.36)	0.9174	122.4 (1.73)	0.2990	0.015	20,318.7 (1.40)	0.3927	129.7 (1.71)	0.1049	0.006
**C57BL/6**	**PBS**	9	50.0 (1.00)	≤0.0001	50.0 (1.00)	0.0006	1.000	50.0 (1.00)	≤0.0001	50.0 (1.00)	<0.0001	1.000
	**VAX**	9	7,036.3 (1.50)		3,638.3 (2.18)		0.517	23,529.0 (1.28)		8,273.2 (1.73)		0.352
	**VAX + ACE**	10	11,313.7 (1.37)	0.3604	1,131.4 (1.69)	0.2346	0.100	27,415.7 (1.33)	0.6867	4,525.5 (1.45)	0.3793	0.165
	**VAX + MEL**	7	8,268.5 (1.47)	0.7731	2,539.8 (1.24)	0.6670	0.307	26,429.2 (1.42)	0.7884	8,063.5 (1.22)	0.9658	0.305

a. Irradiated CO92 or irradiated VAX (Y. pestis CO92 pgm-/pPst-) cells were used as capture antigen at a concentration of 10 µg/ml.
b. Isotype matching of IgG subclass antibodies dependent on inbred mouse strains, such that IgG2a was used for BALB/c and IgG2c was used for C57BL/6 antibody titer determination.
c. P-values reflect the result of post-hoc comparisons under a repeated measures ANOVA model.

Table S11. Total IgG titers determined by ELISA in sera collected from surviving mice 21 days post challenge with fully-virulent *Y. pestis* CO92.

			VAX[a]		
		**n (Survivors)**	**Median**	**Geo Mean (GSE)**	**vs. Pre-challenge (P-Value)[b]**
**BALB/c**	**PBS**	0	n/a	n/a	n/a
	**VAX**	10	12,800	10,159.4 (1.33)	0.0517
	**VAX + ACE**	10	9,051	8,444.9 (1.44)	0.0631
	**VAX + MEL**	10	7,184	5,571.5 (1.30)	0.1910
**C57BL/6**	**PBS**	0	n/a	n/a	n/a
	**VAX**	10	11,314	13,928.8 (1.29)	0.8725
	**VAX + ACE**	10	12,699	12,409.2 (1.54)	0.7023
	**VAX + MEL**	10	12,699	13,928.8 (1.36)	0.9162

a. Irradiated VAX (Y. pestis CO92 pgm-/pPst-) cells were used as capture antigen at a concentration of 10 µg/ml.
b. P-values reflect the result of post-hoc comparisons under a repeated measures ANOVA model.
